# Supplementary material for: The evolutionary advantage of an aromatic clamp in plant family 3 glycoside exo-hydrolases
Source: Nat Commun. 2022 Sep 23;13:5577. doi: 10.1038/s41467-022-33180-5 (PMC9508125; doi:10.1038/s41467-022-33180-5)
Supplement: Supplementary file 1 — Supplementary Information [file 41467_2022_33180_MOESM1_ESM.pdf]

Supplementary Information

The evolutionary advantage of an aromatic clamp in plant family 3 glycoside exo-hydrolases

Sukanya Luang[1,10], Xavier Fernández-Luengo[2,10], Alba Nin-Hill[3,10], Victor A. Streltsov[4], Julian G. Schwerdt[1], Santiago Alonso-Gil[3], James R. Ketudat Cairns[5], Stéphanie Pradeau[6], Sébastien Fort[6], Jean-Didier Maréchal[2], Laura Masgrau[2,7], Carme Rovira[3,8] & Maria Hrmova[1,9,*]

[1]School of Agriculture, Food and Wine, and Waite Research Institute, University of Adelaide, Waite Research Precinct, Glen Osmond, SA, Australia. [2]Department de Química, Universitat Autònoma de Barcelona, Bellaterra, Spain. [3]Department of Química Inorgànica i Orgànica and Institut de Química Teòrica i Computacional, Universitat de Barcelona, Barcelona, Spain. [4]The Florey Institute, University of Melbourne, Victoria, Australia. [5]School of Chemistry, Suranaree University of Technology, Nakhon Ratchasima, Thailand. [6]Université Grenoble Alpes, Centre de Recherches sur les Macromolécules Végétales, Grenoble, France. [7]Institut de Biotecnologia i de Biomedicina, Universitat Autònoma de Barcelona, Bellaterra, Spain. [8]Institució Catalana de Recerca i Estudis Avançats, Barcelona, Spain. [9]Jiangsu Collaborative Innovation Centre for Regional Modern Agriculture and Environmental Protection, School of Life Science, Huaiyin Normal University, Huai'an, China.

[10]These authors contributed equally: Sukanya Luang, Xavier Fernández-Luengo, and Alba Nin-Hill.
[*]Correspondence and requests for materials should be addressed to M.H. (email: maria.hrmova@adelaide.edu.au).

Supplementary Methods

Materials. (1,3;1,6)-β-D-Glucan (laminarin from *Laminaria digitata*), sophorose [β-D-Glc-(1,2)-D-Glc], cellobiose [β-D-Glc-(1,4)-D-Glc], gentiobiose [β-D-Glc-(1,6)-D-Glc], 4-nitrophenyl β-D-glucopyranoside (4NP-Glc), 4-nitrophenyl β-D-cellobioside (4NP-cellobioside) were purchased from Sigma (St. Louis, MO, USA), and barley and Icelandic moss (lichenan) (1,4;1,3)-β-D-glucans, and laminaribiose [β-D-Glc-(1,3)-D-Glc] were from Megazyme (Wicklow, Ireland), and cello-oligosaccharides of the degree of polymerisation (DP) 3-6 and laminari-oligosaccharides of the DP 3-7 were from Seikagaku Kogyo (Tokyo, Japan). Thio-ligand inhibitors to generate complexes included methyl-O-β-thio-sophoroside (G2SG-OMe)[1], 4-nitrophenyl 3^{I}-S-thio-laminaritriose (4NP-G3SG3OG)[2], 4-nitrophenyl 3-thio-laminaribioside (4NP-G3SG) (this work), 4^{I}, 4^{III}, 4^{V}-S-trithiocellohexaose (G4SG4OG4SG4OG4SG)[3], methyl 4-thio-β-cellobioside (G4SG-OMe) (this work) and methyl 6-thio-β-gentiobioside (G6SG-OMe)[1].

Phylogenetic analysis of GH3 members. Sequences of GH3 members, retrieved from public databases GenBank (https://www.ncbi.nlm.nih.gov/genbank), UniProt (https://www.uniprot.org/), Carbohydrate-Active Enzymes (CAZy)[4], and Phytozome13 (https://phytozome-next.jgi.doe.gov/blast-search) databases, are listed in Supplementary Table 1. These sequences were aligned in Muscle[5] and an unrooted phylogenetic tree was constructed in MEGA11[6] by using the Neighbor-Joining method[7] and visualised in FigTree v1.4.3 (https://beast.community/figtree). Individual clades were annotated with

enzyme activities: β-D-glucan glucohydrolases (EC 3.2.1.-), β-D-glucosidases (EC 3.2.1.21), β-N-acetylhexosaminidases (EC 3.2.1.52), β-D-xylosidases (EC 3.2.1.37), and α-L-arabinofuranosidases (EC 3.2.1.55). The tree is drawn to scale, with branch lengths in the same units as those of the evolutionary distances used to infer the phylogenetic tree. The evolutionary distances were computed using the p-distance method[8] and are in the units of the number of amino acid residue differences per site. This analysis involved 62 amino acid sequences. All ambiguous positions were removed for each sequence pair (pairwise deletion option), and we detected a total of 2,127 positions in a final dataset. In some instances, 3D models of entries were built to confirm the structural engagement of domains involved in protein folding and the identity of key residues involved in substrate-product assisted processivity.

Synthesis of 4-Nitrophenyl 3-*S*-β-D-glucopyranosyl-3-thio-β-D-glucopyranoside (4-nitrophenyl 3-thio-laminaribioside). The synthesis proceeded in two steps as follows:

*(i) 4-Nitrophenyl 2,4,6-tri-*O*-acetyl-3-*S*-(2,3,4,6-tetra-*O*-acetyl-β-D-glucopyranosyl)-3-thio-β-D-glucopyranoside*

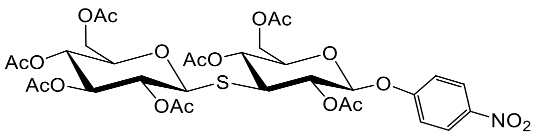

A solution of 2,4,6-tri-*O*-acetyl-3-*S*-(2,3,4,6-tetra-*O*-acetyl-β-D-glucopyranosyl)-3-thio-β-D-glucopyranosyl bromide (400 mg, 0.56 mmol)[9], tetrabutylammonium hydrogen sulfate (190 mg, 0.56 mmol) and 4-nitrophenol (155 mg, 1.12 mmol) in CH_2Cl_2 (5 mL) was mixed with 1M aq NaOH (5 mL). The two-phase reaction mixture was vigorously stirred at room temperature for 3 h after which a total disappearance of the starting material was observed by TLC (solvent AcOEt/EP 1:1). The organic phase was successively washed with cold 1M NaOH (2x20 mL) and water (2x20 mL). The organic phase was dried with anhydrous sodium sulfate, filtered and concentrated. After purification by flash chromatography (EtOAc/EP 1:1), 4-nitrophenyl 2,4,6-tri-*O*-acetyl-3-*S*-(2,3,4,6-tetra-*O*-acetyl-β-D-glucopyranosyl)-3-thio-β-D-glucopyranoside was isolated with 39% yield (170 mg). Analytical data agreed with those reported previously in the literature[10]. MS (ESI+) m/z = 796.06 [M+Na]$^+$; ^{1}H NMR (400 MHz, $CDCl_3$, 298K) δ 8.14 (d, J = 9.2 Hz, 2H), 7.00 (d, J = 9.2 Hz, 2H), 5.32 (dd, J = 10.5, 7.2 Hz, 1H), 5.16 (t, J = 9.2 Hz, 1H), 5.07 – 4.86 (m, 4H), 4.68 (d, J = 10.0 Hz, 1H), 4.25 – 4.14 (2xm, 4H), 3.89 (m, 1H), 3.71 (m, 1H), 3.07 (t, J = 10.5 Hz, 1H), 2.09 – 1.95 (7xs, 21H); ^{13}C NMR (101 MHz, $CDCl_3$, 298K) δ 170.5, 170.3, 170.1, 169.3, 169.2, 169.1, 168.4, 161.1, 143.1, 125.7, 116.4, 99.2, 83.7, 75.6, 74.8, 73.5, 71.9, 70.0, 68.1, 66.3, 62.2, 61.9, 49.6, 20.7, 20.6, 20.5, 20.4.

*(ii) 4-Nitrophenyl 3-*S*-β-D-glucopyranosyl-3-thio-β-D-glucopyranoside (4-nitrophenyl 3-thio-laminaribioside)*

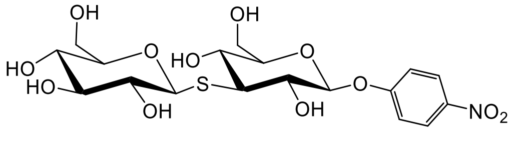

Sodium methylate 1 M (100 µL) was added to a solution of 4-nitrophenyl 2,4,6-tri-*O*-acetyl-3-*S*-(2,3,4,6-tetra-*O*-acetyl-β-D-glucopyranosyl)-3-thio-β-D-glucopyranoside (170 mg, 0.21 mmol) in MeOH/CH_2Cl_2 (1:1 v/v, 5 ml). After 12 h at room temperature, the mixture was neutralised with Amberlite IR 120 H^+ resin, filtered and concentrated. The residue was dissolved with water, and freeze-dried affording 4-nitrophenyl 3-thio-β-laminaribioside (88 mg, 96% yield). MS (ESI+) m/z = 501.98 $[M+Na]^+$; ^{1}H NMR (400 MHz, D_2O, 328K) δ 8.26 (d, 2H, J = 9.2 Hz), 7.26 (d, 2H, J = 8.8 Hz), 5.28 (d, 1H, J = 9.6 Hz), 4.82 (d, 1H, J = 9.6 Hz), 3.95 (m, 2H), 3.84-3.74 (m, 4H), 3.65-3.38 (m, 5H), 3.11 (t, 1H, J = 10.8 Hz); ^{13}C NMR (101 MHz, D_2O, 328K) δ 162.0, 142.9, 126.4, 116.8, 100.8, 84.7, 80.1, 78.9, 77.5, 72.9, 72.2, 69.7, 67.1, 61.1, 61.0, 54.2.

Synthesis of methyl 4-*S*-β-D-glucopyranosyl-4-thio-β-D-glucopyranoside (methyl 4-thio-β-cellobioside)

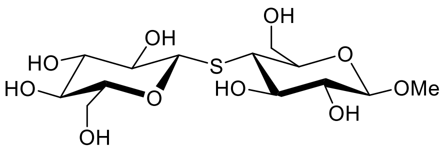

Sodium methylate 1 M (50 µL) was added to a solution of methyl 4-*S*-(2,3,4,6-tetra-*O*-acetyl-β-D-glucopyranosyl)-4-thio-β-D-glucopyranoside[11] (100 mg, 0.18 mmol) in MeOH (5 ml). After 4 h at room temperature, the mixture was neutralised with Amberlite IR 120 H^+ resin, filtered and concentrated. The residue was dissolved with water, and freeze-dried affording methyl 4-thio-β-cellobioside (67 mg, 97% yield). MS (ESI+) m/z 395.04 $[M+Na]^+$; ^{1}H NMR (400 MHz, D_2O, 298K): δ 4.66 (d, 1H, J = 10.0 Hz), 4.38 (d, 1H, J = 8.0 Hz), 4.16 (dd, 1H, J = 2 Hz, J = 12.4Hz), 3.95 (dd, 1H, J = 5.2 Hz, J = 12 Hz), 3.91 (dd, 1H, J = 2.4 Hz, J = 11.6 Hz), 3.72 (m, 2H), 3.63-3.30 (m, 9H), 2.88 (t, 1H, J = 10.8 Hz); ^{13}C NMR (101 MHz, D_2O, 298K) δ 102.9, 83.9, 79.8, 77.2, 76.4, 74.3, 72.9, 72.5, 69.4, 61.4, 60.8, 57.1, 47.1.

Supplementary Note 1

Physico-chemical properties of Trp286 and Trp434 mutants. To define physico-chemical properties such as pH optima and thermostability, we compared the pH optima of mutants to those of the WT enzyme (pH optimum 5.25)[12] and observed that the relative activities of single or double mutants W286F, W286H, W286Y, W434A, W434F, W434H, and W434Y, W286F/W434F, W286F/W434A remained essentially unchanged or changed little (Supplementary Fig. 1a). The exceptions were the W286H and W286F/W434A mutants, which shifted their pH optima to the more alkaline regions of 6.8 and 5.8, respectively, while other mutants retained similar temperature tolerance, except the double W286F/W434F mutant, which was by about 4 °C more temperature-sensitive (Supplementary Fig. 1b).

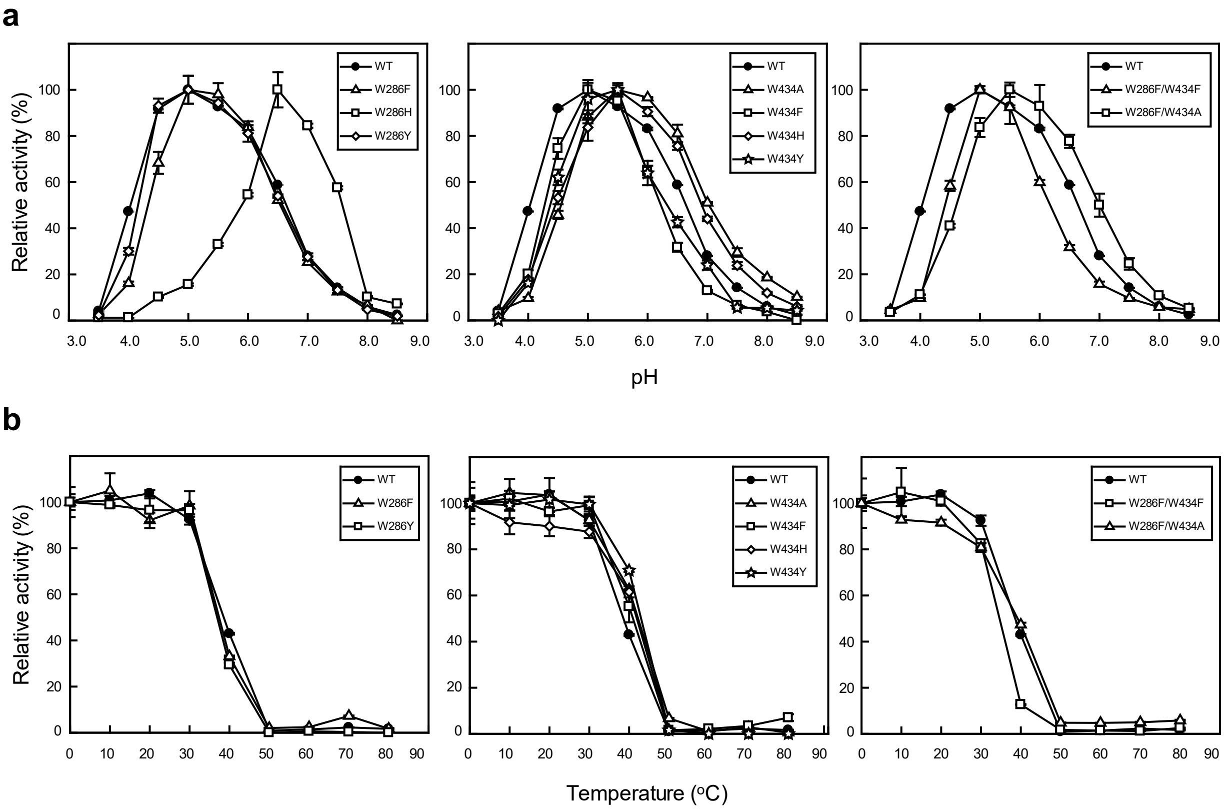

Supplementary Fig. 1. Dependence of pH or temperature for hydrolysis of 4NP-Glc catalysed by wild-type (WT) and mutant HvExoI.

Enzyme activities (expressed as relative activities) were determined at 410 nm at the indicated pH (**a**) or temperature (**b**) values. Data are presented as mean values from three measurements with standard deviations.

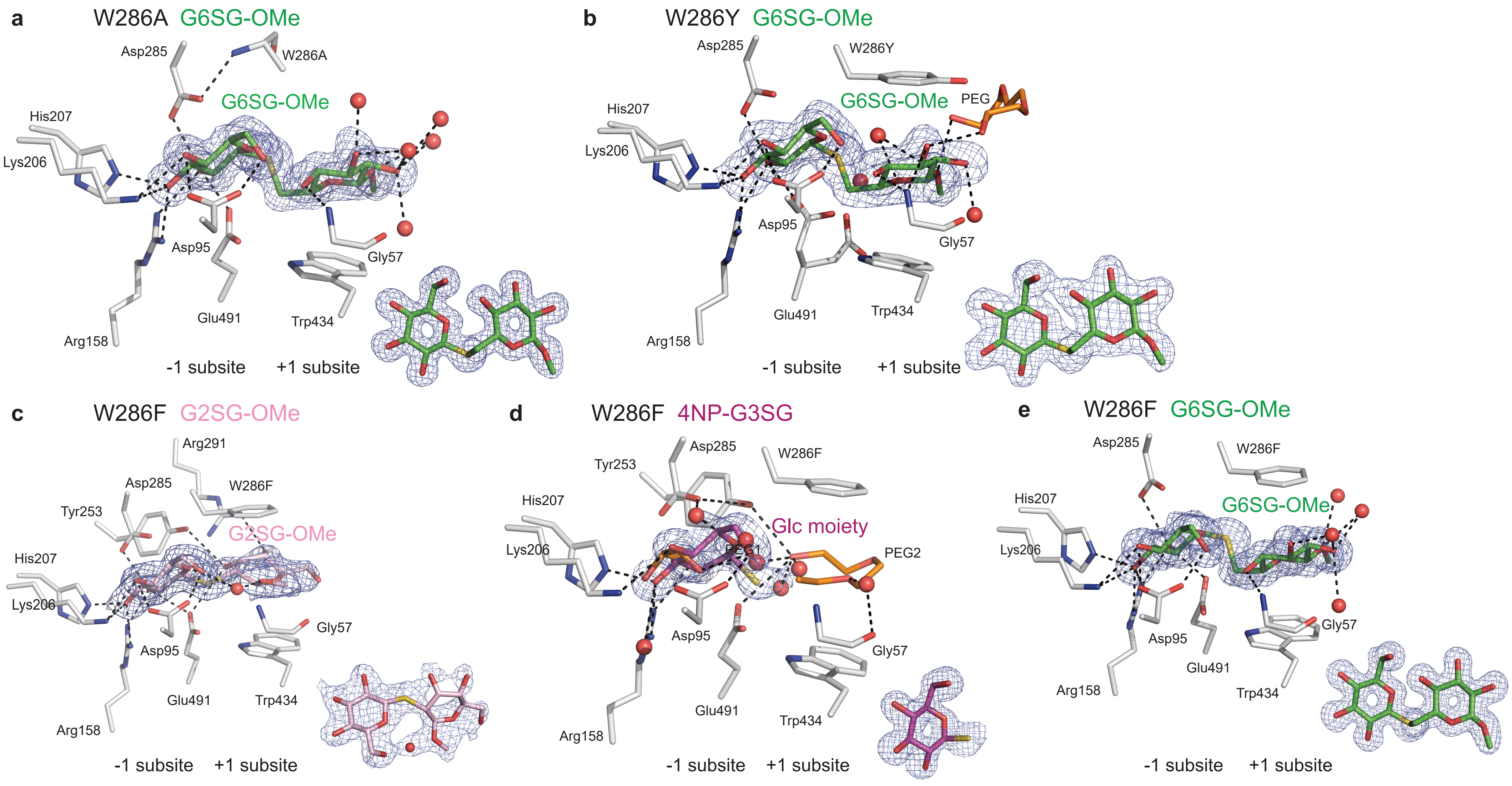

Supplementary Fig. 2. Thio-saccharide analogues are bound in the active site of the HvExoI Trp286 mutants.

a Thio-gentiobioside (G6SG-OMe; green) in W286A, and **b** in W286Y are bound across the -1 and +1 subsites; **c** thio-sophoroside (G2SG-OMe; pink); **d** thio-laminaribioside (4NP-G3SG; magenta); and **e** thio-gentiobioside (G6SG-OMe; green) in W286F are bound across the -1 and +1 subsites. In the complex with 4NP-G3SG, only the saccharide moiety at the -1 subsite is modelled. Panels **a** to **d** contain derived $2m|F_o|-D|F_c|$ difference electron density maps (blue) of thio-analogues (also shown as insets rotated by around -90° *via* x-axes relative to main panels) contoured at 1.0 σ levels. Polyethylene glycol (PEG) and water molecules are shown in cpk orange sticks and red spheres, respectively. Distances at separations within 3.3 Å are shown in dashed lines. Ligand designations above structural images indicate thio-analogues that were perfused in crystals.

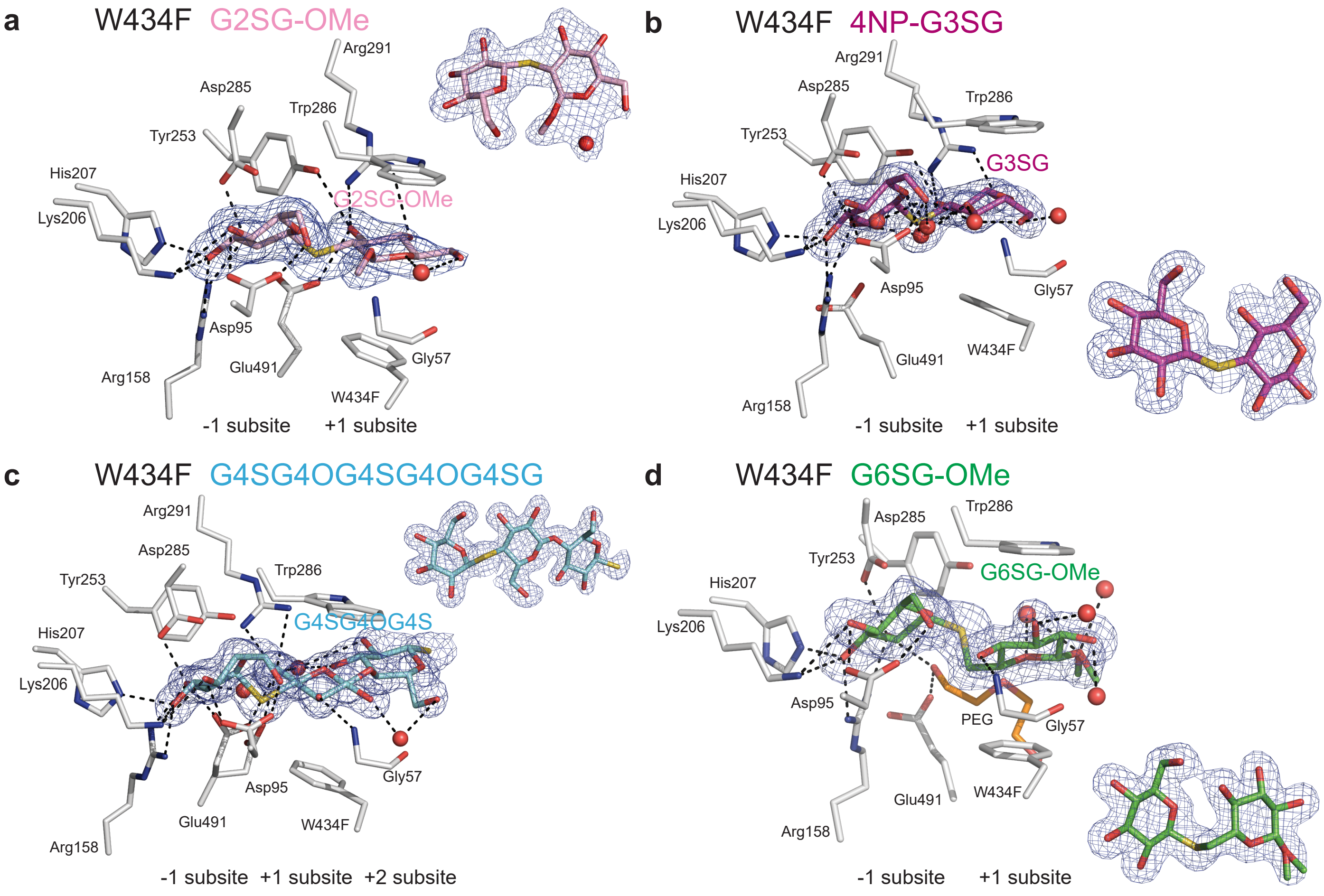

Supplementary Fig. 3. Thio-saccharide analogues are bound in the active site of the HvExoI W434F mutant.

a Thio-sophoroside (G2SG-OMe, pink); **b** thio-laminaribioside (4NP-G3SG, magenta); **c** thio-cellotrioside (G4SG4OGS; cyan); and **d** thio-gentiobioside (G6SG-OMe, green) moieties are bound across the -1 to +2 subsites. Panels **a** to **d** contain derived 2m|F_o|-D|F_c| difference electron density maps (blue) of thio-analogues (also shown as insets rotated by around -90º *via* x-axes relative to main panels) contoured at 1.0 σ levels. Glycerol (Gol) or polyethylene glycol (PEG), and water molecules are shown in cpk orange sticks and red spheres, respectively. Distances at separations within 3.3 Å are shown in dashed lines. Ligand designations above structural images indicate thio-analogues that were perfused in crystals.

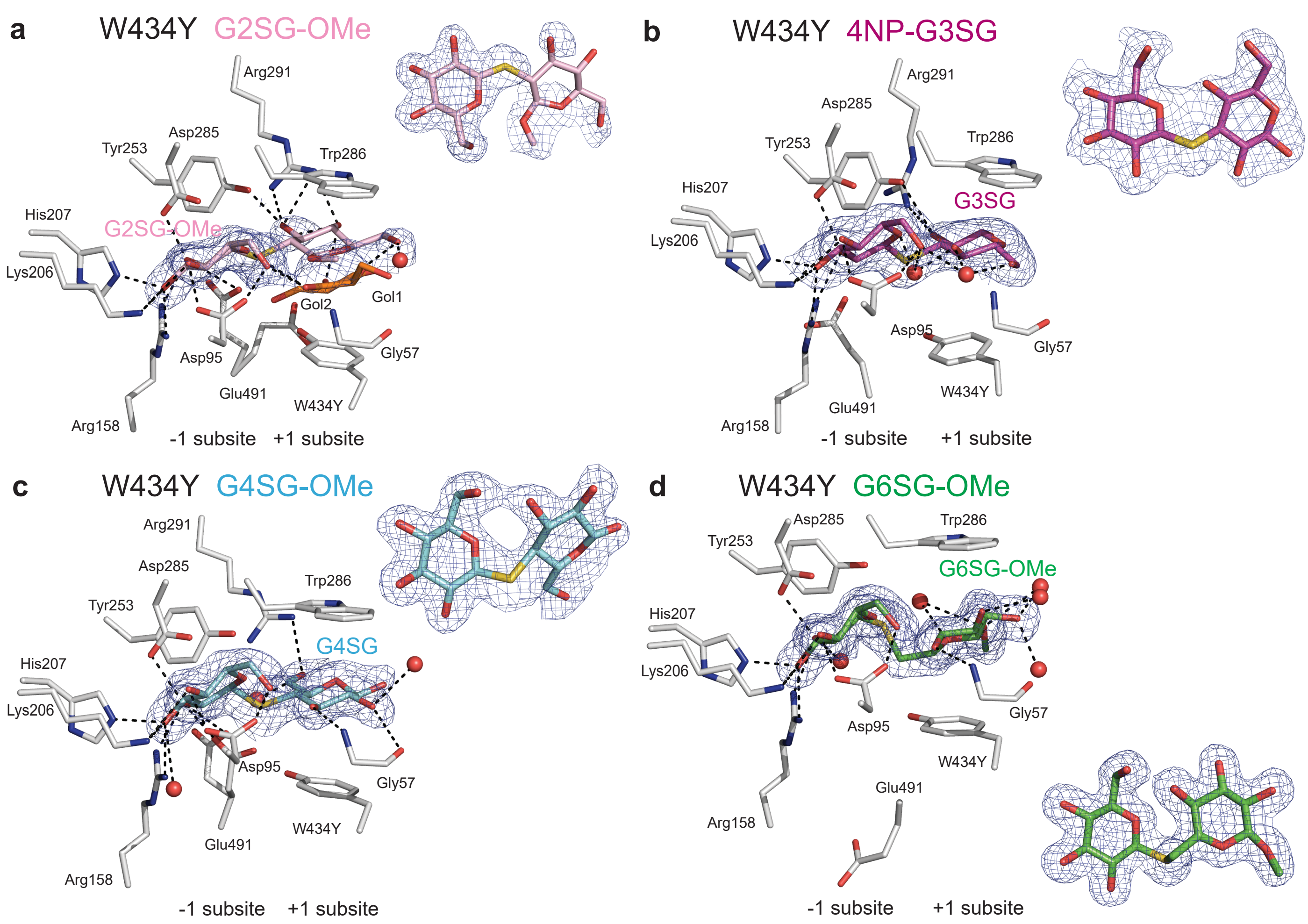

Supplementary Fig. 4. Thio-saccharide analogues are bound in the active site of the HvExoI W434Y mutant.

a Thio-sophoroside (G2SG-OMe, pink); **b** thio-laminaribioside (4NP-G3SG, magenta); **c** thio-cellobioside (G4SG; cyan); and **d** thio-gentiobioside (G6SG-OMe, green) moieties are bound across the -1 to +1 subsites. Panels **a** to **d** contain derived 2m$|F_o|$-$D|F_c|$ difference electron density maps (blue) of thio-analogues (also shown as insets rotated by around -90° *via* x-axes relative to main panels) contoured at 1.0 σ levels. Glycerol (Gol) or polyethylene glycol (PEG), and water molecules are shown in cpk orange sticks and red spheres, respectively. Distances at separations within 3.3 Å are shown in dashed lines. Ligand designations above structural images indicate thio-analogues that were perfused in crystals.

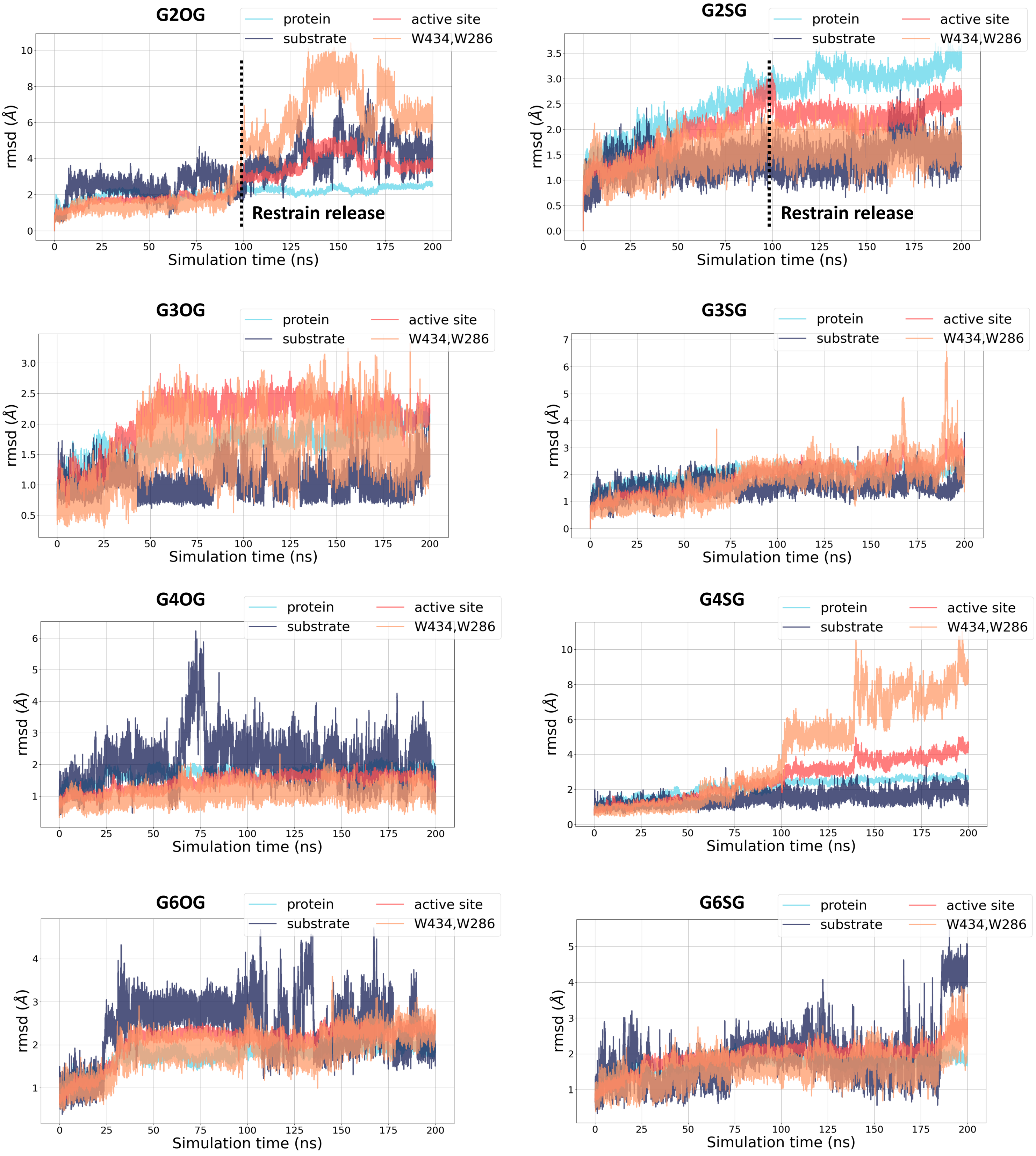

Supplementary Fig. 5. NTV cMD simulations of HvExoI in complex with G2OG or G2SG, G3OG or G3SG, G4OG or G4SG, and G6OG or G6SG.

Evolution of RMSD values of protein backbones (light blue), active site residues (red), the Trp434 and Trp286 residues (orange), and O- and S-linked oligosaccharides (dark blue).

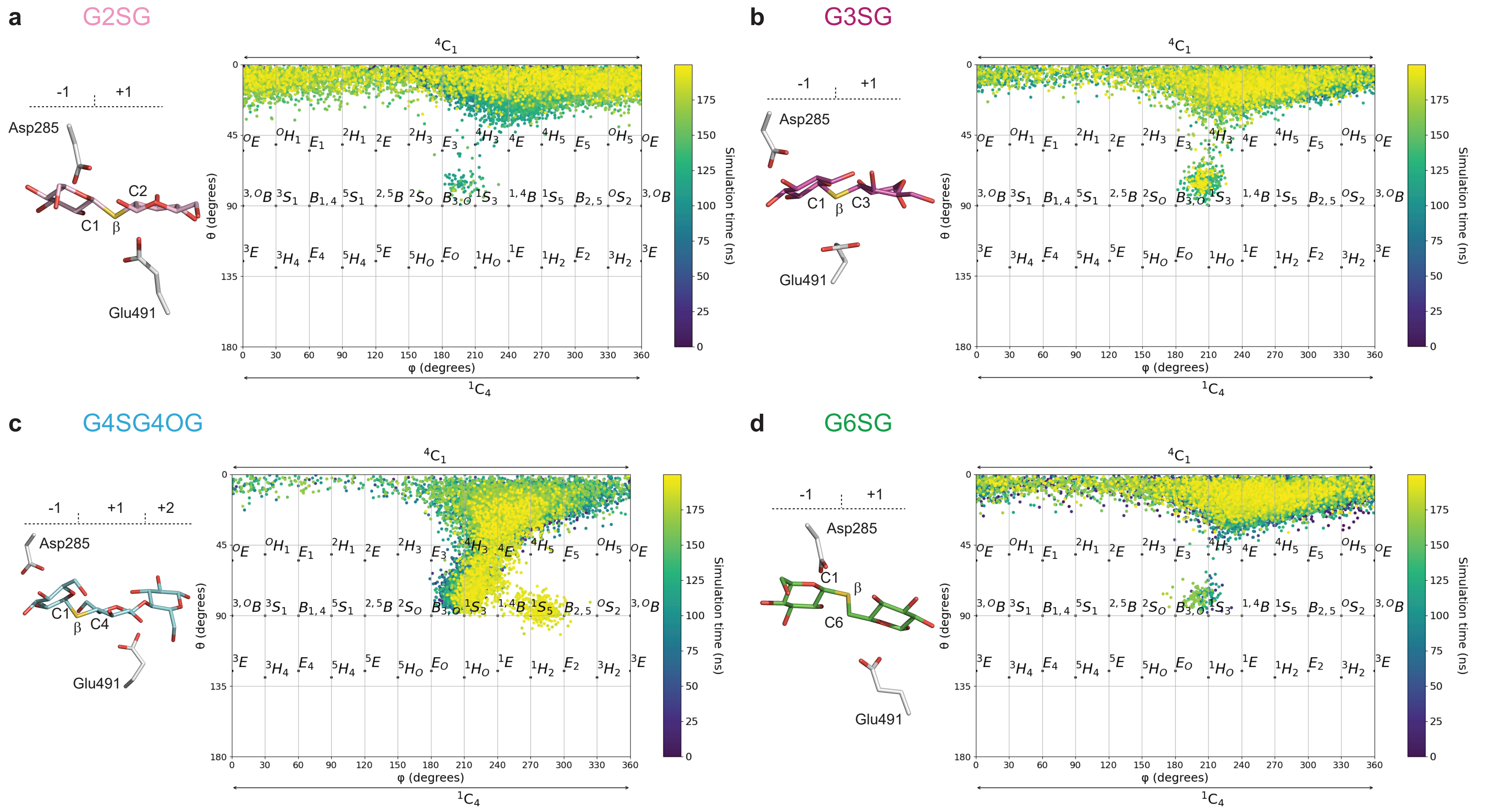

Supplementary Fig. 6. Behaviour of S-linked oligosaccharides bound in the active site of HvExoI calculated by cMD simulations plotted as a function of θ and φ puckering coordinates.

a Thio-sophorose (G2SG; pink); **b** thio-laminaribiose (G3SG; magenta); **c** 4I-S-thio-cellotriose (G4SG4OG; cyan); and **d** thio-gentiobiose (G6SG; green) ligands are bound at the -1 to +2 subsites (colour gradients from purple at 0 ns to yellow at 200 ns in Mercator projections are indicated. The β-D-glucopyranose moieties at the -1 to +2 subsites, and dispositions of Asp285 and Glu491 catalytic residues are also shown.

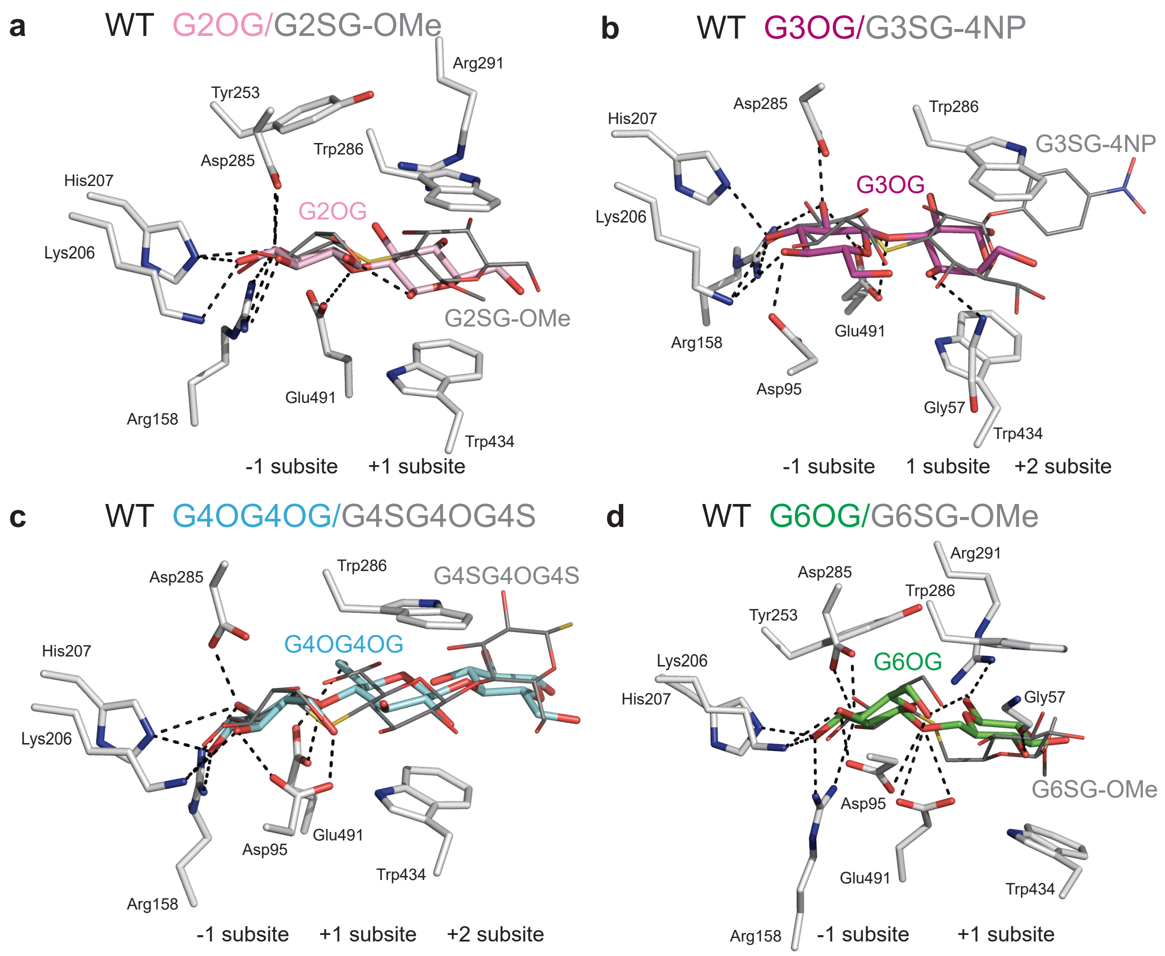

Supplementary Fig. 7. Binding modes of O-linked oligosaccharides (based on thio-analogue complexes) attached in the HvExoI active site, calculated *via* cMD.

a Sophorose (G2OG; pink); **b** laminaribiose (G3OG; magenta); **c** cellotriose (G4OG4OG); cyan); and **d** gentiobiose (G6OG; green) are bound in the -1 and +2 subsites. Complexes of WT with 4NP-G3SG, G4SG4OG4S, and G6SG-OMe thio-analogues, and W286F in complex with G2SG-OMe (indicated in grey cpk lines and grey letters) were used to predict the binding modes of O-linked oligosaccharides.

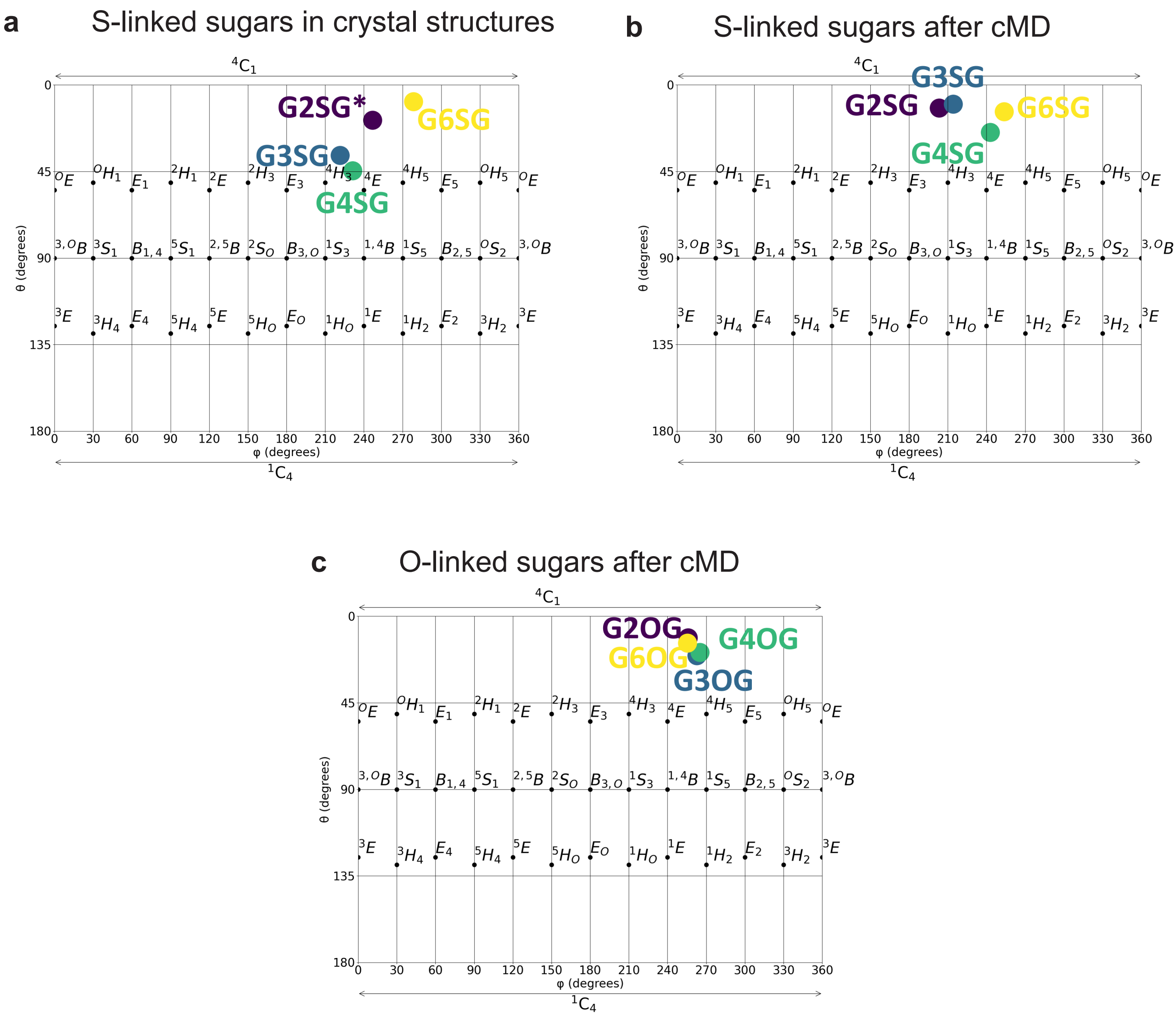

Supplementary Fig. 8. Conformational behaviour (Mercator projections) of Glc moieties at the -1 subsite of S- and O-linked oligosaccharides, plotted as a function of θ and φ puckering coordinates.

a Thio-analogues in the crystal structures of WT HvExoI (*G2SG taken from W434A); **b** Thio-analogues based on crystal structures after cMD; **c** O-linked oligosaccharides based on crystal structures after cMD simulations.

Conformational FEL map of the -1 subsite Glc of G3OG

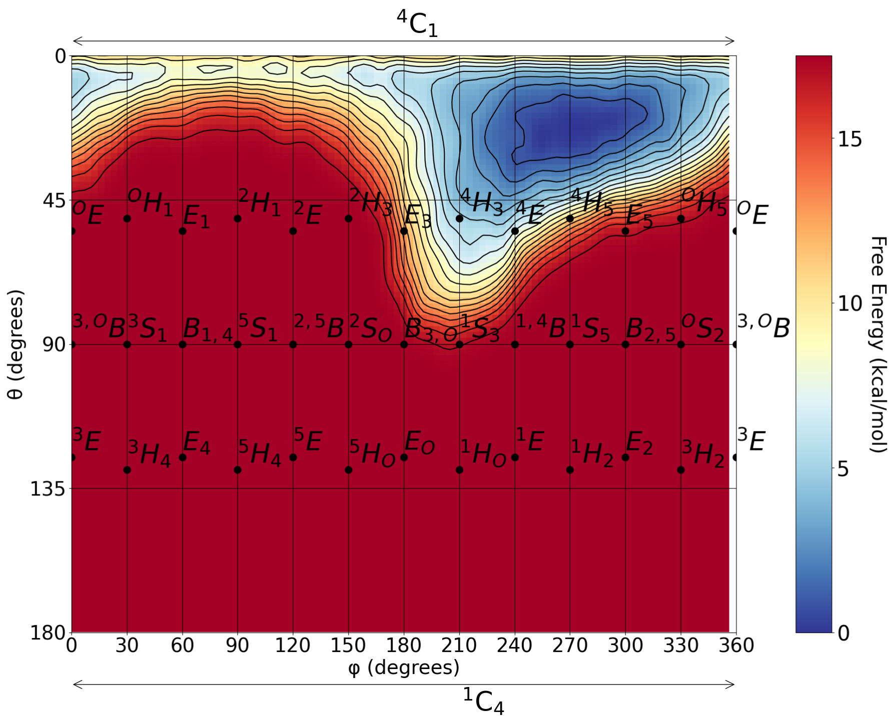

Supplementary Fig. 9. Conformational FEL map of the Glc moiety of G3OG at the -1 subsite in the active site of WT HvExoI, plotted as a function of θ and φ puckering coordinates.

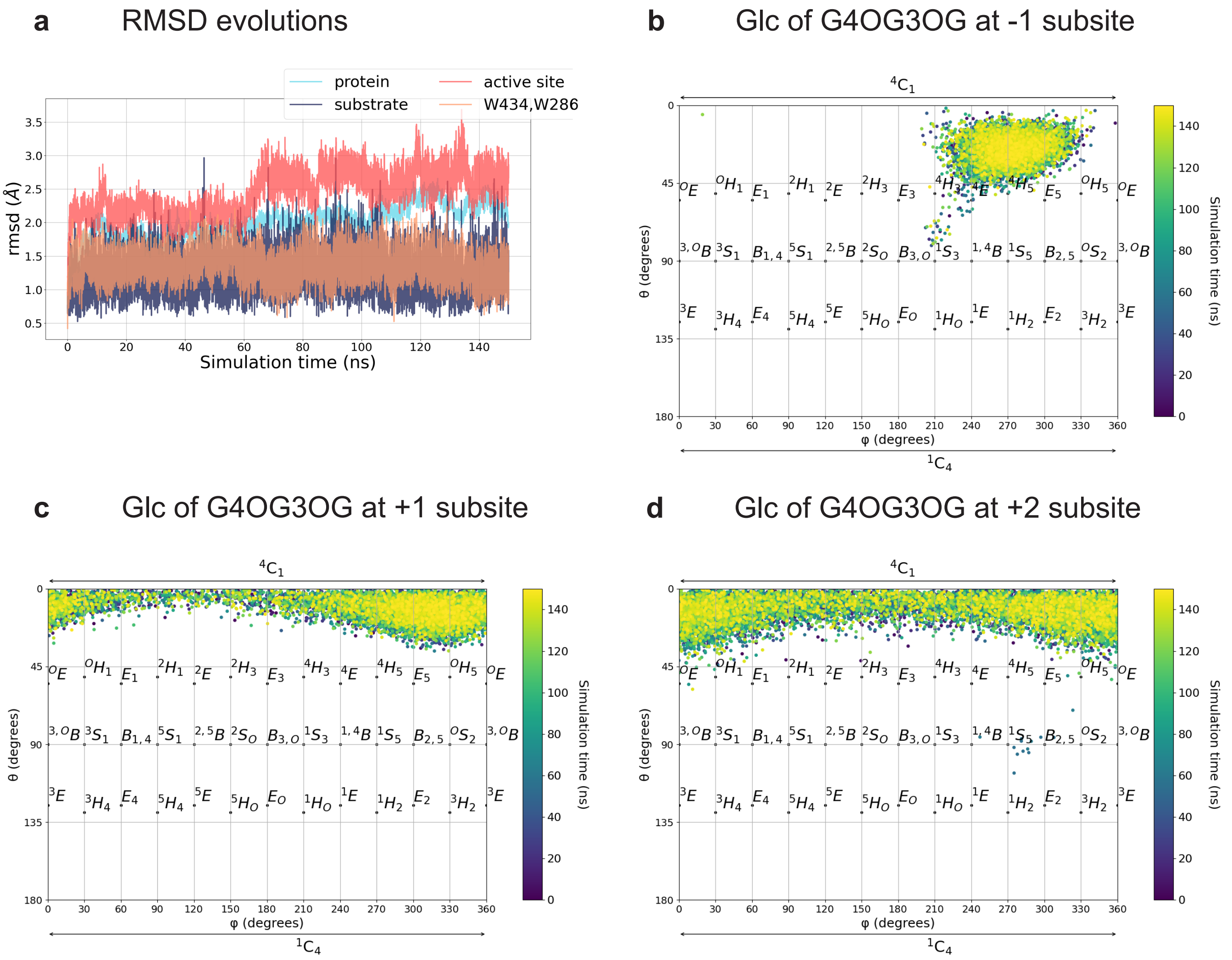

Supplementary Fig. 10. NTV cMD simulation of HvExoI in complex with the natural substrate G4OG3OG and its conformational behaviour.

a Evolution of RMSD values of the protein backbone (light blue), active site residues (red), G4OG3OG (dark blue), and the Trp434 and Trp286 residues (orange). **b-d** Analysis of the puckering ring coordinates of Glc moieties of G4OG3OG bound in the -1 to +2 subsites along the classical MD trajectory. Colour gradients from purple at 0 ns to yellow at 150 ns in Mercator projections, plotted as a function of θ and φ pucker ing coordinates are shown.

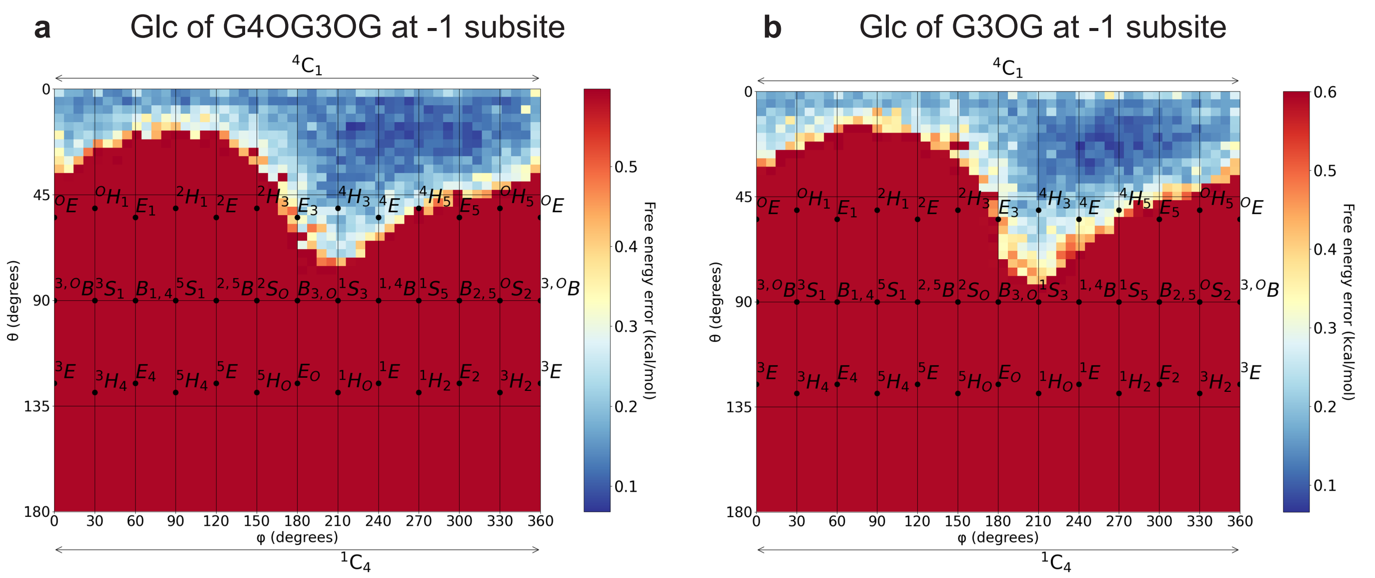

Supplementary Fig. 11. Free energy error of conformational FEL maps of Glc molecules in the -1 subsites of WT HvExoI, plotted as a function of θ and φ puckering coordinates.

a G4OG3OG substrate; **b** G3OG product originating from G4OG3OG.

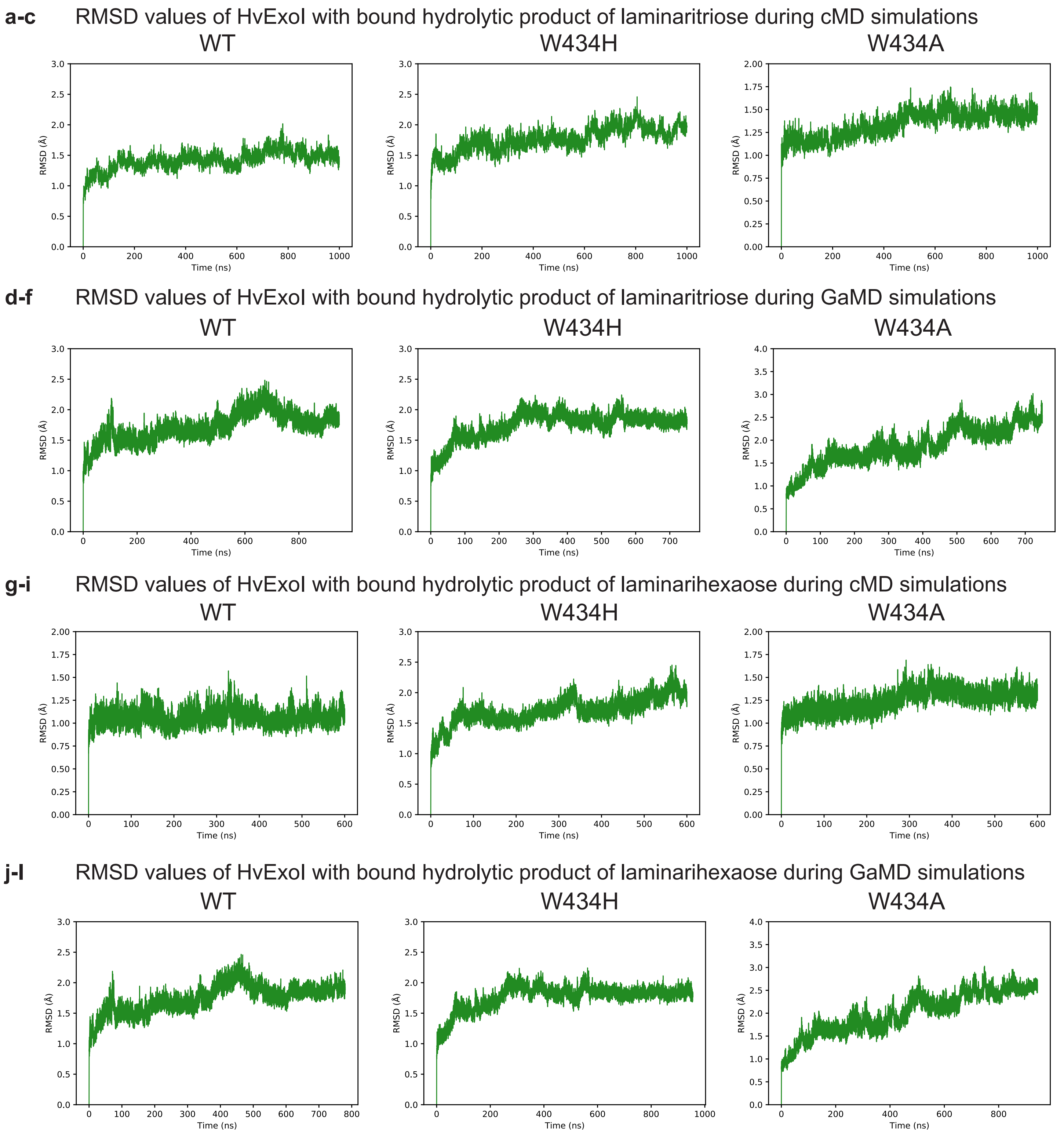

Supplementary Fig. 12. RMSD values of HvExoI with bound hydrolytic products of laminaritriose and laminarihexaose during cMD and GaMD simulations.

a-c RMSD values in WT, W434H and W434A with bound hydrolytic products of laminaritriose during cMD simulations. **d-f** RMSD values in WT, W434H and W434A bound hydrolytic products of laminaritriose during GaMD simulations. **g-i** RMSD values in WT, W434H and W434A with bound hydrolytic products of laminarihexaose during cMD simulations. **j-l** RMSD values in WT, W434H and W434A with bound hydrolytic products of laminarihexaose during GaMD simulations.

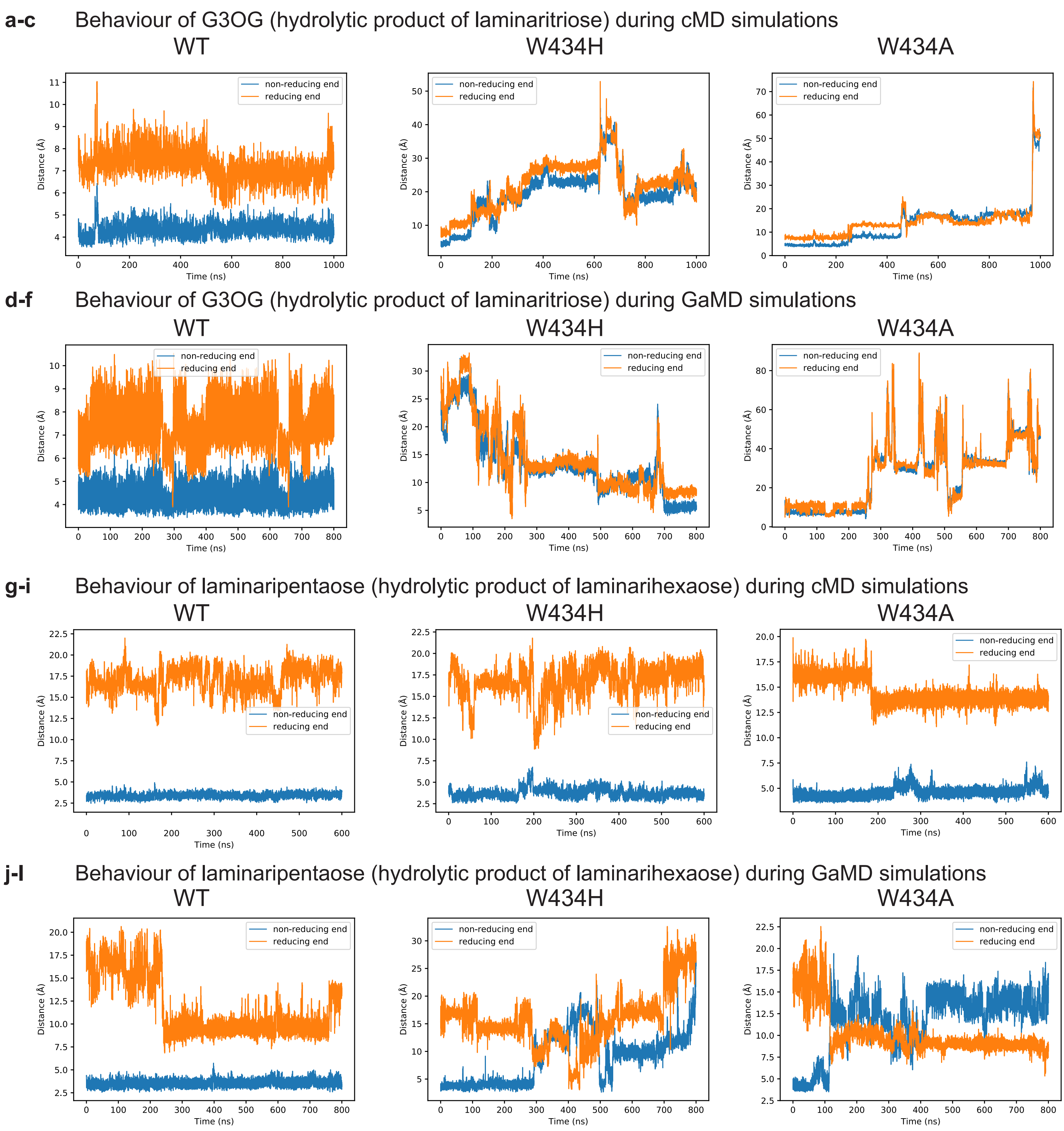

Supplementary Fig. 13. The behaviour of G3OG and laminaripentaose, hydrolytic products of laminaritriose and laminarihexaose, respectively, bound to HvExoI, explored by cMD and GaMD simulations. Distances between reducing and non-reducing ends of products at the +1 and +2 subsites were used as a metric to analyse their stability.

a-c The behaviour of G3OG during cMD simulations. G3OG is stable in the +1 and +2 subsites in WT, while in W434H and W434A, G3OG diffuses to bulk solvent. **d-f** The behaviour of G3OG during GaMD simulations. G3OG is stable in the +1 and +2 subsites in WT, but in W434H G3OG shows an inverse diffusion from bulk solvent to the space formed by the +1 and +2 subsites, and in W434A, G3OG diffuses to bulk solvent. **g-i** The behaviour of laminaripentaose during cMD simulations. WT and W434H and W434A hold laminaripentaose in the space formed by the +1 and +2 subsites. **j-l** The behaviour of laminaripentaose during GaMD simulations. Laminaripentaose is stable in the +1 and +2 subsites in WT, while in W434H and W434A laminaripentaose shows inverted re-attachment to the +1 and +2 subsites. Mutations of Trp434 reduce the stability of laminaripentaose in the +1 and +2 subsites; longer oligosaccharides are more stable than shorter ones.

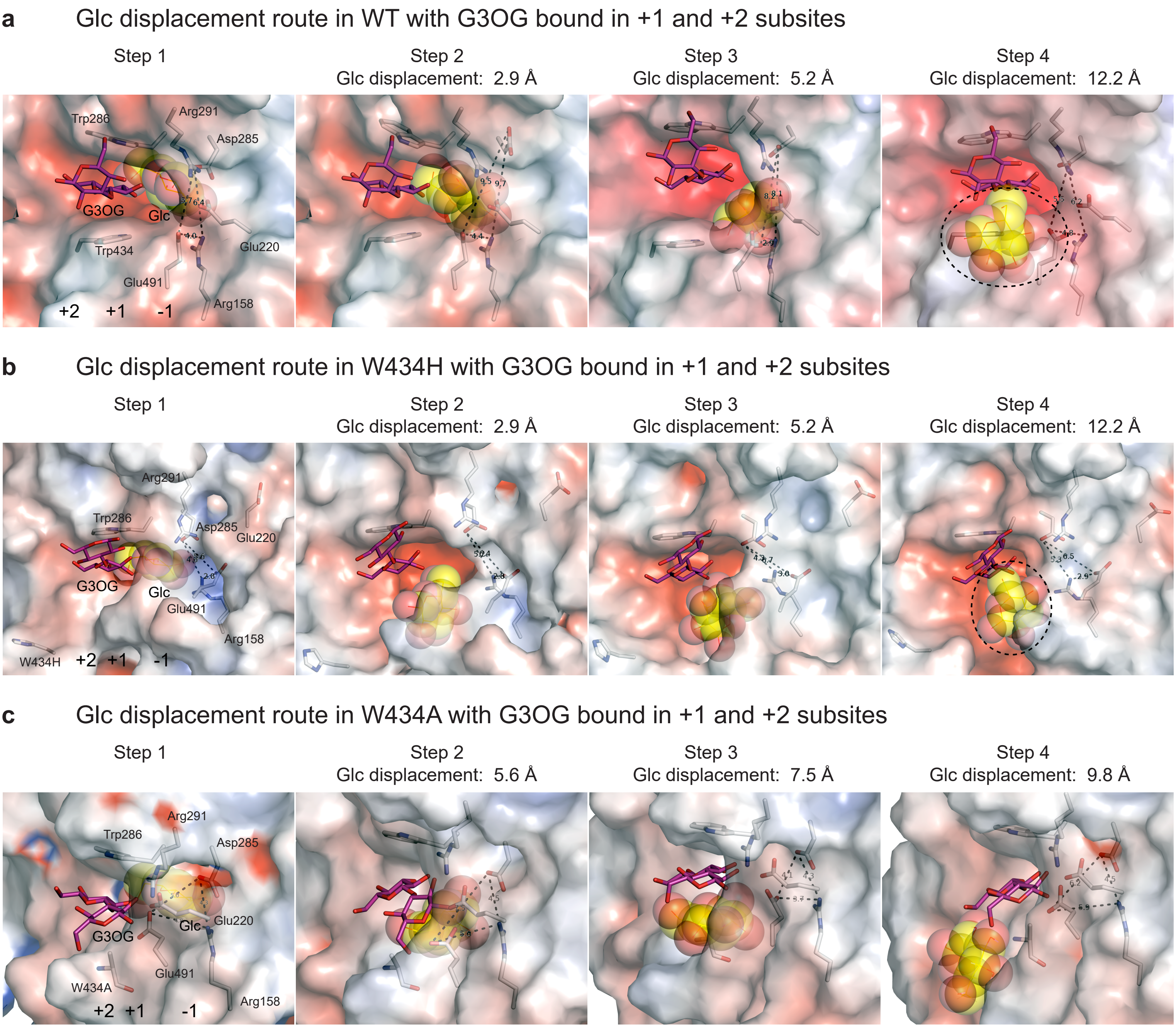

Supplementary Fig. 14. Glc displacement route in WT, and the W434H and W434A mutants of HvExoI with Glc and G3OG, calculated through cMD, and GPathFinder.

a WT; **b** W434H; **c** W434A – Four steps along Glc displacement routes based on converged structures in complex with Glc (-1 subsite; carbons in cpk yellow lines, and spheres) and G3OG (+1 and +2 subsites and bulk solvent; carbons: magenta sticks) obtained by docking. Selected residues (carbons: atomic sticks), and positions of Glc, and G3OG are indicated. Glc in the -1 subsite is separated from the lateral cavity that is evolving during Glc egress by the participation of Arg158-Asp285-Glu491 toll-like barrier (triangles in dashed lines; distances indicated in Å). Surface morphologies of structures are coloured by electrostatic potentials: white, neutral; blue, +5 kT·e^{-1}; red, -5 kT·e^{-1}. Separations in Å between the positions of C1 carbons of Glc molecules, as they move from the -1 subsites in initial and final structures are indicated. The lateral cavity (panels **a**-**b**; indicated in black dotted ellipsoids) forms transiently, is partly exposed to bulk solvent and facilitates Glc displacement. In panel **c**, Glc is displaced to bulk solvent through the opening formed by the W434A mutation.

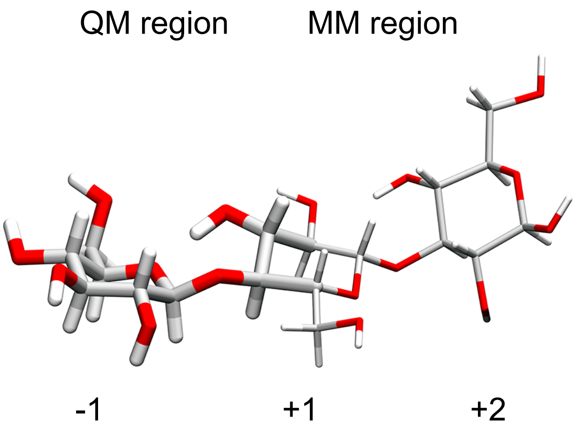

Supplementary Fig. 15. The G4OG3OG substrate, which was used for the conformational Free Energy Landscape (FEL) simulation in the active site (-1 to +2 subsites) of the WT enzyme, consisted of QM and MM regions. The QM region (atoms) included the Glc molecule positioned in the -1 subsite and the half-ring of Glc placed in the +1 subsite including two monovalent pseudopotentials to saturate the QM region (shown in sticks). The MM region (atoms) included the other half-ring of Glc placed in the +1 subsite and the third Glc moiety placed in the +2 subsite (shown in lines). For the sake of clarity, the other MM atoms corresponding to the protein and water molecules are not shown.

Supplementary Table 1 The list of 62 GH3 entries analysed for phylogeny (*cf.* Fig. 1c).

	Enzyme	Activity	Species	Accession number (Genbank/ UniProt/ Phytozome 13)
1.	Hv Exo I	β-D-glucan exohydrolase isoenzyme ExoI	*Hordeum vulgare*	AF102868
2	Hv Exo II	β-D-glucan exohydrolase isoenzyme ExoII	*Hordeum vulgare*	U46003
3.	Zm Exo II	exhydrolase II	*Zea mays*	AAD28356.1
4.	Nt Exo	β-D-glucan exohydrolase	*Nicotiana tabacum*	BAA33065.1
5.	Tm bglu	β-D-glucosidase	*Tropaeolum majus*	CAA07070.1
6.	At Exo I	β-D-glucan exohydrolase-like protein	*Arabidopsis thaliana*	Q9SD73
7.	At Exo II	β-D-glucan exohydrolase-like protein	*Arabidopsis thaliana*	Q9SD69
8.	At Exo III	β-D-glucan exohydrolase-like protein	*Arabidopsis thaliana*	Q9SD72
9.	Sf bglu	glucan 1,3-β-glucosidase	*Sphagnum fallax*	0201s0003.1.p
10.	Cp bglu	β-D-glucosidase	*Ceratodon purpureus*	CepurGG1.1G085400.1.p
11.	Pp bglu	exo-1,3-β-glucosidase	*Physcomitrella patens*	Pp3c26_8580V3.1.p
12.	Sm bglu	β-D-glucosidase	*Selaginella moellendorffii*	157260
13.	Cr bglu	β-D-glucosidase	*Ceratopteris richardii*	Ceric.31G056500.2.p
14.	Cf GH3	GH3 hydrolase	*Cephalotus follicularis*	A0A1Q3BSS3
15.	Aa bglu	β-D-glucosidase	*Arabis alpina*	A0A087GVX0
16.	Es bglu	β-D-glucosidase	*Eutrema salsugineum*	V4LTP2
17.	Fv bglu	β-D-glucosidase	*Fragaria vesca*	XP_004296697.1
18.	La unpro	uncharacterised protein	*Lupinus angustifolius*	XP_019430114.1
19.	Rs unpro	uncharacterised protein	*Raphanus sativus*	018443128.1
20.	At bglu	β-D-glucosidase	*Amborella trichopoda*	W1PPJ1
21.	At unpro I	uncharacterised protein	*Amborella trichopoda*	W1NE16_9
22.	At unpro II	uncharacterised protein	*Amborella trichopoda*	W1NE06
23.	Ps bglu I	β-D-glucosidase	*Picea sitchensis*	E0Z601_2
24.	Ps bglu II	β-D-glucosidase	*Picea sitchensis*	A9NUD1_27
25.	Mp bglu I	β-D-glucosidase	*Marchantia polymorpha*	A0A176WE76
26.	Mp bglu II	β-D-glucosidase	*Marchantia polymorpha*	Mapoly0014s0035.1.p
27.	Mp bglu III	β-D-glucosidase	*Marchantia polymorpha*	Mapoly0214s0013.3.p
28.	Tb bglu	β-D-glucosidase	*Thermoanaerobacter brockii*	P96090
29.	Te xyl-ara	xylosidase/arabinosidase	*Thermoanaerobacter ethanolicus*	AAF43783.1
30.	Hv bxyl	β-D-xylosidase	*Hordeum vulgare*	AAK38482.1
31.	Nc bglu	β-D-glucosidase	*Neurospora crassa*	XP_958209.2
32.	Mp bxyl I	β-D-xylosidase	*Marchantia polymorpha*	Mapoly0071s0042.1.p
33.	Mp bxyl II	β-D-xylosidase	*Marchantia polymorpha*	Mapoly0124s0035.1.p
34.	Mp bxyl III	β-D-xylosidase	*Marchantia polymorpha*	Mapoly0106s0041.1.p
35.	Mp bxyl IV	β-D-xylosidase	*Marchantia polymorpha*	Mapoly0069s0044.1.p
36.	Rf GH3	GH3 hydrolase	*Ruminococcus flavefaciens*	CAB51937.1
37.	Hj bxyl	β-D-xyloosidase precursor	*Hypocrea jecorina*	Q92458

38.	Cg bglu	β-D-glucosidase	*Cellulomonas gilvus*	BAA03152.1
39.	Ci bglu	β-D-glucosidase	*Coccidioides immitis*	U87805
40.	Sc bglu	β-D-glucosidase	*Saccharomycopsis fibuligera*	M22476
41.	Gg ave	avenacinase	*Gaeumannomyces graminis*	AAB09777.1
42.	Wa bglu	β-D-glucosidase	*Wickerhamomyces anomalus*	P06835
43.	Hj GH3	GH3 hydrolase	*Hypocrea jecorina*	XP_006964076.1

44.	Tb gln-glc	glucan glucohydrolase	*Thermobispora bispora*	AAB36835.1
45.	Tn bglu	β-D-glucosidase	*Thermotoga neapolitana*	Q60038
46.	Se GH3	GH3 hydrolase	*Saccharopolyspora erythraea*	WP_011873141.1
47.	Ar bglu	β-D-glucosidase	*Agrobacterium rhizogenes*	WP_142888755.1
48.	Pb GH3	GH3 hydrolase	*Prevotella bryantii*	WP_006282898.1
49.	Km bglu	β-D-glucosidase	*Kluyveromyces marxianus*	P07337.1

50.	Pp hexo	hexosaminidase	*Pseudoalteromonas piscicida*	P48823
51.	Ec bglu	β-D-glucosidase	*Escherichia coli*	WP_000871504.1
52.	Dc bglu	β-D-glucosidase	*Dickeya chrysanthemi*	AAA80156.1
53.	Ni bglu	β-D-glucosidase precursor	*Niveispirillum irakense*	AAF21799.1
54.	St ac-glc-am	β-N-acetylglucosaminidase	*Streptomyces thermoviolaceus*	BAA32403.1
55.	Dd bglu	β-D-glucosidase	*Dictyostelium discoideum*	AAA74233.1
56.	Bs bglu	β-D-glucosidase	*Bacillus sp.*	BAA36161.1
57.	Ps ac-glc-ami	β-N-acetylglucosaminidase	*Paenibacillus sp.*	A0A5S8WF56
58.	Cs GH3	GH3 hydrolase	*Cyanobacterium stanieri*	AFZ46311.1
59.	Cs bglu	β-D-glucosidase	*Cyanobacterium sp. HL-69*	AUC61062.1
60.	Cr GH3	GH3 protein	*Chlamydomonas reinhardtii*	Cre03.g200655.t1.1

61.	Cb bglu	β-D-glucosidase	*Chloroflexi bacterium*	A0A2N2MB45
62.	Xc bglu	β-D-glucosidase	*Xanthomonas campestris*	B0RTT5_XANCB

Supplementary Table 2 Relative rates of hydrolysis of poly- and oligosaccharides, and aryl-glycosides by WT and mutant HvExoI.

Substrate	Activity (µmole/min per mg protein)										
	WT	W286A	W286H	W286F	W286Y	W434A	W434H	W434F	W434Y	W286F/ W434F	W286F/ W434A
Polysaccharides											
(1,3;1,6)-β-D-Glucan (laminarin; 3:2) [a]	54.4 ± 0.5 [b]	nd [c]	nm [d]	1.1 ± 0.1	14.2 ± 1.3	nd	0.7 ± 0.02	7.8 ± 0.4	0.7 ± 0.03	0.2 ± 0.01	0.1 ± 0.003
(1,4;1,3)-β-D-Glucan (barley glucan; 2.3-2.7:1) [a]	5.4 ± 0.3	nd	nm	nd	1.2 ± 0.02	nd	0.04 ± 0.01	nd	nd	0.03 ± 0.001	nd
(1,4;1,3)-β-D-Glucan (lichenin; 2:1) [a]	0.8 ± 0.1	nd	nm	nd	nd	nd	0.03 ± 0.01	nd	nd	0.03 ± 0.001	nd
Oligosaccharides											
Sophorose	6.0 ± 0.1	nd	nd	0.6 ± 0.01	12.5 ± 0.2	nd	0.6 ± 0.1	6.6 ± 0.4	0.8 ± 0.1	0.3 ± 0.03	0.02 ± 0.001
Laminaribiose	1.3 ± 0.01	nd	nd	0.6 ± 0.04	13.9 ± 0.1	0.5 ± 0.03	0.8 ± 0.04	14.9 ± 1.7	1.1 ± 0.1	0.5 ± 0.1	0.1 ± 0.001
Laminaritriose	12.4 ± 0.3	nd	nm	1.3 ± 0.01	14.1 ± 0.4	0.9 ± 0.1	1.5 ± 0.04	18.4 ± 1.4	1.4 ± 0.2	0.5 ± 0.04	0.1 ± 0.002
Laminaritetraose	12.3 ± 0.5	nd	nm	1.1 ± 0.1	13.8 ± 0.1	1.1 ± 0.1	1.4 ± 0.1	15.0 ± 1.5	1.3 ± 0.1	0.4 ± 0.04	0.2 ± 0.01
Laminaripentaose	11.7 ± 0.5	nd	nm	1.0 ± 0.01	14.8 ± 0.4	1.0 ± 0.1	1.5 ± 0.1	14.6 ± 0.4	1.5 ± 0.1	0.4 ± 0.04	0.2 ± 0.02
Laminarihexaose	12.2 ± 0.1	nd	nm	1.1 ± 0.03	15.8 ± 0.1	1.0 ± 0.02	1.5 ± 0.1	15.8 ± 1.0	1.4 ± 0.1	0.5 ± 0.1	0.1 ± 0.004
Laminariheptaose	11.0 ± 0.8	nd	nm	1.2 ± 0.1	14.5 ± 2.0	1.0 ± 0.1	1.6 ± 0.1	13.5 ± 0.4	1.3 ± 0.1	0.5 ± 0.04	0.1 ± 0.004
Cellobiose	4.6 ± 0.2	nd	nd	0.1 ± 0.01	3.2 ± 0.1	nd	0.1 ± 0.01	0.4 ± 0.1	0.1 ± 0.001	0.01 ± 0.001	0.01 ± 0.001
Cellotriose	8.8 ± 0.4	nd	nm	0.1 ± 0.01	5.6 ± 0.1	nd	0.3 ± 0.03	1.2 ± 0.3	0.1 ± 0.02	0.02 ± 0.002	0.01 ± 0.001
Cellotetraose	8.5 ± 0.7	nd	nm	0.1 ± 0.01	6.3 ± 1.1	nd	0.2 ± 0.01	1.9 ± 0.1	0.1 ± 0.01	0.02 ± 0.001	0.1 ± 0.01
Cellopentaose	8.4 ± 0.4	nd	nm	0.1 ± 0.01	5.8 ± 0.2	nd	0.2 ± 0.01	1.7 ± 0.1	0.1 ± 0.002	0.02 ± 0.001	0.01 ± 0.0003
Cellohexaose	9.1 ± 0.2	nd	nm	0.1 ± 0.01	7.0 ± 0.1	nd	0.3 ± 0.02	1.9 ± 0.1	0.1 ± 0.01	0.01 ± 0.001	0.01 ± 0.0002
Gentiobiose	4.7 ± 0.4	nd	nd	0.4 ± 0.03	7.6 ± 0.8	nd	0.3 ± 0.02	1.2 ± 0.3	0.1 ± 0.01	0.01 ± 0.001	0.002 ± 0.0002
Aryl-glycosides											
4NP-Glc	12.4 ± 0.8	nd	nd	0.9 ± 0.01	19.6 ± 0.01	6.1 ± 0.1	2.4 ± 0.2	5.0 ± 0.01	1.2 ± 0.1	0.5 ± 0.02	1.8 ± 0.03
4NP-cellobioside	0.9 ± 0.01	nd	nm	nd	nd	nd	0.04 ± 0.001	nd	nd	nd	nd

[a] Main types linkages and the ratio of linkage type (13).

[b] Standard deviations are given after ± signs.

[c] nd, (Activity) not detected.

[d] nm, (Experiment) not measured.

Supplementary Table 3 First- and second-order apparent kinetic constants of WT and mutant HvExoI [a].

Substrate	WT	W286F	W286Y	W434A	W434H	W434F	W434Y	W286F/ W434F	W286F/ W434A
(1,3;1,6)-β-D-Glucan (laminarin)									
K_M (mM)	0.2 ± 0.02 [b]	0.2 ± 0.01	0.1 ± 0.01	nm [c]	0.3 ± 0.02	0.2 ± 0.01	0.1 ± 0.01	0.2 ± 0.02	nm
k_{cat} (s^{-1})	42.1 ± 7.6	4.5 ± 0.3	30.3 ± 2.4	nm	8.5 ± 0.9	8.0 ± 0.7	0.8 ± 0.1	0.4 ± 0.1	nm
k_{cat}/K_M ($mM^{-1}\cdot s^{-1}$)	234.0 ± 41.0	27.4 ± 2.2	252.7 ± 38.0	nm	33.4 ± 2.3	52.7 ± 6.4	7.6 ± 1.3	1.8 ± 0.2	nm
(1,4;1,3)-β-D-Glucan (barley glucan)									
K_M (mM)	0.04 ± 0.004	nm	0.1 ± 0.01	nm	nm	nm	nm	0.02 ± 0.002	nm
k_{cat} (s^{-1})	14.1 ± 1.5	nm	15.0 ± 1.1	nm	nm	nm	nm	0.3 ± 0.03	nm
k_{cat}/K_M ($mM^{-1}\cdot s^{-1}$)	365.5 ± 58.0		208.8 ± 1.5	nm	nm	nm	nm	16.8 ± 1.7	nm
Laminaribiose									
K_M (mM)	1.3 ± 0.12	0.4 ± 0.04	0.4 ± 0.04	2.1 ± 0.2	1.7 ± 0.4	1.7 ± 0.2	0.6 ± 0.1	0.2 ± 0.02	0.7 ± 0.2
k_{cat} (s^{-1})	54.2 ± 6.9	2.6 ± 0.2	20.7 ± 1.7	2.6 ± 0.2	51.8 ± 2.0	17.1 ± 1.6	2.0 ± 0.1	0.5 ± 0.04	0.2 ± 0.02
k_{cat}/K_M ($mM^{-1}\cdot s^{-1}$)	41.2 ± 5.6	5.8 ± 0.4	52.6 ± 1.5	1.3 ± 0.1	31.3 ± 7.3	9.9 ± 1.2	3.3 ± 0.3	3.0 ± 0.6	0.2 ± 0.03
Cellobiose									
K_M (mM)	1.2 ± 0.1	nm	1.4 ± 0.1	nm	2.8 ± 0.1	1.2 ± 0.2	nm	1.5 ± 0.3	nm
k_{cat} (s^{-1})	4.4 ± 0.1	nm	4.5 ± 0.3	nm	1.2 ± 0.1	0.6 ± 0.03	nm	0.02 ± 0.001	nm
k_{cat}/K_M ($mM^{-1}\cdot s^{-1}$)	3.7 ± 0.2	nm	3.2 ± 0.4	nm	0.4 ± 0.01	0.5 ± 0.02	nm	0.01 ± 0.001	nm
4NP-Glc									
K_M (mM)	2.0 ± 0.15	2.4 ± 0.3	2.0 ± 0.1	0.5 ± 0.1	2.2 ± 0.2	0.6 ± 0.04	0.3 ± 0.03	0.6 ± 0.1	0.7 ± 0.04
k_{cat} (s^{-1})	27.7 ± 2.5	1.9 ± 0.1	28.8 ± 2.9	9.5 ± 0.7	7.7 ± 0.6	6.6 ± 0.7	1.2 ± 0.1	0.7 ± 0.02	1.9 ± 0.1
k_{cat}/K_M ($mM^{-1}\cdot s^{-1}$)	13.9 ±0.6	0.8 ± 0.01	14.6 ± 1.5	17.6 ± 1.8	3.5 ± 0.4	12.7 ± 2.1	3.5 ± 0.4	1.2 ± 0.2	2.5 ± 0.2

[a] Parameters not measured with W286A and W286H.

[b] Standard deviations are given after the ± signs.

[c] nm, (Experiment) not measured.

Supplementary Table 4 Inhibition parameters of native, and recombinant WT and mutant HvExoI by thio-analogues.

Inhibitor	Native HvExoI	Recombinant HvExoI [a]						
		WT	W286F	W286Y	W434A	W434H	W434F	W434Y
G2SG-OMe								
K_i (M)	2 x 10^{-3}	4 x 10^{-3}	nm [c]	~13 x 10^{-3}	nm	nm	nm	nm
ΔG ($kJ \cdot mol^{-1}$) [b]	-15.7	-13.9						
4NP-G3SG								
K_i (M)	0.7 x 10^{-3}	0.7 x 10^{-3}	0.7 x 10^{-3}	2 x 10^{-3}	1.5 x 10^{-3}	2.5 x 10^{-3}	1.1 x 10^{-3}	2 x 10^{-3}
ΔG ($kJ \cdot mol^{-1}$)	-18.3	-18.3	-18.3	-15.7	-16.4	-15.1	-17.2	-15.7
G4SG-OMe								
K_i (M)	1.0 x 10^{-3}	1.0 x 10^{-3}	2 x 10^{-3}	2.2 x 10^{-3}	2.2 x 10^{-3}	1.7 x 10^{-3}	3.4 x 10^{-3}	4.2 x 10^{-3}
ΔG ($kJ \cdot mol^{-1}$)	-17.4	-17.4	-15.7	-15.4	-15.4	-16.1	-14.3	-13.8
G6SG-OMe								
K_i (M)	0.1 x 10^{-3}	0.2 x 10^{-3}	0.2 x 10^{-3}	0.3 x 10^{-3}	~16 x 10^{-3}	0.8 x 10^{-3}	0.6 x 10^{-3}	0.3 x 10^{-3}
ΔG ($kJ \cdot mol^{-1}$)	-23.2	-21.3	-21.5	-20.4		-18.0	-18.7	-20.4

[a] K_i values are expressed in one or two significant digits.

[b] Calculated according to $\Delta G = -RT \ln [1/K_i]$ (14).

[c] nm, (Experiment) not measured.

Supplementary Table 5 List of crystal structures of WT and mutant HvExoI in complex with thio-analogues and Glc.

PDB accession	HvExoI	Ligand diffused [a, b]	Ligand bound [c] Subsite -1	Subsite +1	Reference
3WLO	WT	Glc	Glc	Glc	(1)
6MD6	WT	G2SG-OMe	G2SG-OMe (+1 to +2 subsites) [d]		(1)
6JG2	WT	4NP-G3SG	4NP-G3SG (-1 to +2 subsites) [d]		This work
6JG1	WT	(G4SG4O)$_3$	G4SG4OG4S (-1 to +2 subsites) [d]		This work
3WLP	WT	G6SG-OMe	G6SG-OMe ^{rot}		(1)
6JG6	W286A	G6SG-OMe	G6SG-OMe ^{rot}		This work
6JG7	W286F	G2SG-OMe	G2SG-OMe		This work
6JGA	W286F	4NP-G3SG	G3S	disordered [e]	This work
6JGB	W286F	G6SG-OMe	G6SG-OMe ^{rot}		This work
6JGC	W286Y	Glc	Glc	Glc	This work
6JGD	W286Y	G6SG-OMe	G6SG-OMe ^{rot}		This work
6KUF	W434A	Glc	Glc	disordered [e]	This work
6JGE	W434A	G2SG-OMe	G2SG-OMe		This work
6L1J	W434A	4NP-G3SG3OG	G3SG		This work
6LBB	W434A	(G4SG4O)$_3$	G4SG4OG4S (-1 to +2 subsites) [d]		This work
6K6V	W434A	G6SG-OMe	G6SG-OMe ^{unrot}		This work
6JGG	W434F	G2SG-OMe	G2SG-OMe		This work
6LC5	W434F	4NP-G3SG	G3SG		This work
6JGK	W434F	(G4SG4O)$_3$	G4SG4OG4S (-1 to +2 subsites) [d]		This work
6LBV	W434F	G6SG-OMe	G6SG-OMe ^{rot}		This work
6JGL	W434H	G2SG-OMe	G2SG-OMe		This work
6JGN	W434H	4NP-G3SG	G3SG		This work
6JGO	W434H	G4SG-OMe	G4SG		This work
6JGP	W434H	G6SG-OMe	G6SG-OMe ^{rot}		This work
6JGQ	W434Y	G2SG-OMe	G2SG-OMe		This work
6JGR	W434Y	4NP-G3SG	G3SG		This work
6JGS	W434Y	G4SG-OMe	G4SG		This work
6JGT	W434Y	G6SG-OMe	G6SG-OMe ^{rot}		This work

[a] Diffused ligands are methyl 2-thio-β-sophoroside (G2SG-OMe), 4-nitrophenyl 3-thio-laminaribioside (4NP-G3SG), 4-nitrophenyl 3^{I}-S-thio-laminaritriose (4NP-G3SG3OG), 4^{I}, 4^{III}, 4^{V}-S-tri-thio-cellohexaose (G4SG4O)$_3$, methyl 4-thio-β-cellobioside (G4SG-OMe), and methyl 6-thio-β-gentiobioside (G6SG-OMe).

[b] Diffusion time between 5-720 min.

[c] PEG and glycerol molecules are not assigned as bound ligands.

[d] Sugar moieties present at the +1 to +2 subsites (G2SG-OMe) and the -1 to +2 subsites (G4SG4OG4S).

[e] Sugar moiety in the +1 subsite is disordered.

[rot/unrot] The reducing-end Glc moiety of G6SG-OMe is rotated (or un-rotated) in the +1 subsite, compared to the position of the reducing-end Glc moiety in WT or in-solution gentiobiose.

Supplementary Table 6 Data collection and refinement statistics of crystal structures of WT and mutant HvExoI in complex with thio-analogues, and Glc.

Parameter	WT	
	4NP-G3SG	$(G4SG4O)_3$
X-Ray source		
Wavelength	1.5418	0.9537
Space group	$P4_32_12$	$P4_32_12$
Unit cell dimensions	a=b=100.18; c=182.51; α=β=γ=90°	a=b=99.76; c=183.17; α=β=γ=90°
Resolution range	87.82-2.00	87.61-1.78
Highest shell (Å)	2.05-2.00	1.83-1.78
Reflections	58,669	84,211
Redundancy	24	29
R_{merge} [a] (%)	9.6 (83.5) [b]	9.2 (80.0) [b]
<I/σ(I)>	27.5 (4.3) [b]	73.0 (3.8) [b]
Completeness (%)	97.2 (94.4) [b]	99.9 (98.9) [b]
$CC_{1/2}$	0.995	0.999
Refinement statistics		
R_{work} (%)	18.0 (38.8) [b]	14.0 (16.1) [b]
R_{free} [c] (%)	22.6 (42.4) [b]	17.0 (20.7) [b]
No. of atoms (protein)	4,600	4,628
(sugar ligands)	24	35
(waters)	356	545
Mean B-value (Å)	36.31	21.35
Rms bonds (Å), angles (°)	0.023; 1.969	0.030; 2.643
Overall coordinate error [d] (Å)	0.137	0.074
Ramachandran plot statistics		
Residues	602	602
Most favoured region	89.4	90.0
Allowed regions	10.2	9.6
Disallowed region	0.4	0.4

[a] $R_{merge} = 100\ [\sum(I_i-\langle I\rangle)^2/\sum I_i^2]$, summed over all independent reflections.

[b] For the highest resolution shell in parenthesis.

[c] Represents approximately 5% of the data.

[d] Based on R_{free}.

Parameter	W286A
	G6SG-OMe
X-Ray source	
Wavelength	0.9537
Space group	$P4_32_12$
Unit cell dimensions	a=b=100.53; c=182.07; α=β=γ=90°
Resolution range	88.01-1.55
Highest shell (Å)	1.59-1.55
Reflections	124,377
Redundancy	20
R_{merge} [a] (%)	6.6 (81.8) [b]
<I/σ(I)>	43.4 (1.0) [b]
Completeness (%)	96.8 (89.8) [b]
$CC_{1/2}$	0.998
Refinement statistics	
R_{work} (%)	16.0 (29.5) [b]
R_{free} [c] (%)	18.7 (30.7) [b]
No. of atoms (protein)	4,604
(sugar ligands)	24
(waters)	760
Mean B-value (Å)	25.31
Rms bonds (Å), angles (°)	0.030; 2.742
Overall coordinate error [d] (Å)	0.063
Ramachandran plot statistics	
Residues	606
Most favoured region	89.8
Allowed regions	9.8
Disallowed region	0.4

[a] $R_{merge} = 100\ [\sum(I_i - \langle I \rangle)^2 / \sum I_i^2]$, summed over all independent reflections.

[b] For the highest resolution shell in parenthesis.

[c] Represents approximately 5% of the data.

[d] Based on R_{free}.

Parameter	W286F		
	G2SG-OMe	4NP-G3SG	G6SG-OMe
X-Ray source			
Wavelength	0.9537	0.9537	0.9537
Space group	$P4_32_12$	$P4_32_12$	$P4_32_12$
Unit cell dimensions	a=b=99.97; c=182.50; α=β=γ=90°	a=b=100.46; c=181.68; α=β=γ=90°	a=b=100.83; c=181.09; α=β=γ=90°
Resolution range	87.68-2.16	87.91-1.47	88.09-1.47
Highest shell (Å)	2.21-2.16	1.51-1.47	1.51-1.47
Reflections	47,914	149,209	149,649
Redundancy	29	24	29
R_{merge} [a] (%)	13.3 (85.7) [b]	6.6 (81.0) [b]	7.3 (82.3) [b]
$\langle I/\sigma(I)\rangle$	46.7 (3.8) [b]	50.8 (1.1) [b]	30.7 (0.7) [b]
Completeness (%)	99.8 (96.9) [b]	99.6 (99.6) [b]	99.5 (98.3) [b]
$CC_{1/2}$	0.998	0.998	0.996
Refinement statistics			
R_{work} (%)	16.3 (22.6) [b]	15.1 (29.2) [b]	15.2 (29.0) [b]
R_{free} [c] (%)	20.8 (29.0) [b]	17.5 (31.1) [b]	17.2 (28.4) [b]
No. of atoms (protein)	4,609	4,619	4,627
(sugar ligands)	24	12	24
(waters)	430	824	630
Mean B-value (Å)	24.01	23.10	21.39
Rms bonds (Å), angles (°)	0.019; 2.029	0.032; 2.906	0.032; 3.067
Overall coordinate error [d] (Å)	0.150	0.051	0.050
Ramachandran plot statistics			
Residues	606	606	606
Most favoured region	88.8	90.4	90.6
Allowed regions	10.8	9.2	9.2
Disallowed region	0.4	0.4	0.2

[a] $R_{merge} = 100\ [\sum(I_i - \langle I\rangle)^2/\sum I_i^2]$, summed over all independent reflections.

[b] For the highest resolution shell in parenthesis.

[c] Represents approximately 5% of the data.

[d] Based on R_{free}.

Parameter	W286Y	
	Glc	G6SG-OMe
X-Ray source		
Wavelength	0.9537	0.9537
Space group	$P4_32_12$	$P4_32_12$
Unit cell dimensions	a=b=100.92; c=181.50; α=β=γ=90°	a=b=101.23; c=180.33; α=β=γ=90°
Resolution range	48.62-2.54	88.27-2.13
Highest shell (Å)	2.61-2.54	2.19-2.13
Reflections	30,121	50,439
Redundancy	29	29
R_{merge} [a] (%)	24.6 (92.6) [b]	20.4 (90.1) [b]
$\langle I/\sigma(I) \rangle$	37.4 (4.0) [b]	23.0 (6.2) [b]
Completeness (%)	99.9 (98.7) [b]	99.9 (99.3) [b]
$CC_{1/2}$	0.997	0.998
Refinement statistics		
R_{work} (%)	13.4 (14.6) [b]	13.7 (15.2) [b]
R_{free} [c] (%)	19.2 (21.6) [b]	17.3 (19.5) [b]
No. of atoms (protein)	4,598	4,608
(sugar ligands)	24	24
(waters)	301	399
Mean B-value (Å)	37.65	21.31
Rms bonds (Å), angles (°)	0.017; 1.943	0.025; 2.261
Overall coordinate error [d] (Å)	0.205	0.120
Ramachandran plot statistics		
Residues	602	603
Most favoured region	89.9	89.3
Allowed regions	9.9	10.5
Disallowed region	0.2	0.2

[a] $R_{merge} = 100\ [\sum(I_i - \langle I \rangle)^2 / \sum I_i^2]$, summed over all independent reflections.

[b] For the highest resolution shell in parenthesis.

[c] Represents approximately 5% of the data.

[d] Not refined.

[e] Based on R_{free}.

Parameter	W434A				
	Glc	G2SG-OMe	4NP-G3SG3OG	$(G4SG4O)_3$	G6SG-OMe
X-Ray source					
Wavelength	0.9537	0.9537	0.9537	0.9537	0.9615
Space group	$P4_32_12$	$P4_32_12$	$P4_32_12$	$P4_32_12$	$P4_32_12$
Unit cell dimensions	a=b=100.69; c=182.39; α=β=γ=90º	a=b=100.83; c=180.99; α=β=γ=90º	a=b=100.82; c=182.33; α=β=γ=90º	a=b=100.57; c=182.31; α=β=γ=90º	a=b=100.52; c=182.08; α=β=γ=90º
Resolution range	88.15-1.90	88.08-1.92	88.23-1.80	88.06-1.80	88.00-1.98
Highest shell (Å)	1.95-1.90	1.97-1.92	1.85-1.80	1.85-1.80	2.03-1.98
Reflections	70,151	67,919	83,048	82,693	62,175
Redundancy	24.5	29	26.3	24.1	29
R_{merge} [a] (%)	8.1 (82.7) [b]	14.1 (86.3) [b]	5.5 (81.2) [b]	6.7 (81.9) [b]	8.2 (82.3) [b]
$\langle I/\sigma(I)\rangle$	29.8 (1.1) [b]	28.5 (6.2) [b]	51.6 (2.2) [b]	43.2 (2.0) [b]	38.7 (6.0) [b]
Completeness (%)	99.2 (95.9) [b]	99.9 (99.2) [b]	99.8 (99.8) [b]	99.8 (98.6) [b]	97.0 (98.2) [b]
$CC_{1/2}$	0.996	0.995	0.999	0.998	0.997
Refinement statistics					
R_{work} (%)	15.2 (29.1) [b]	13.2 (16.0) [b]	14.2 (21.0) [b]	14.0 (22.6) [b]	16.2 (16.6) [b]
R_{free} [c] (%)	19.1 (33.9) [b]	16.5 (19.5) [b]	16.9 (25.5) [b]	16.8 (26.3) [b]	20.9 (22.0) [b]
No. of atoms (protein)	5,448	4,612	4,620	4,627	4,625
(sugar ligands)	12	24	23	34	24
(waters)	730	613	725	769	776
Mean B-value (Å)	25.95	20.83	25.06	25.58	29.38
Rms bonds (Å), angles (º)	0.022; 1.982	0.028; 2.444	0.030; 2.477	0.030; 2.552	0.025; 1.818
Overall coordinate error [d]	0.099	0.092	0.080	0.080	0.126
Ramachandran plot statistics					
Residues	606	606	606	606	606
Most favoured region	90.2	90.6	90.0	90.0	89.4
Allowed regions	9.4	9.0	9.6	9.6	10.2
Disallowed region	0.4	0.4	0.4	0.4	0.4

[a] $R_{merge} = 100\ [\sum(I_i - \langle I\rangle)^2/\sum I_i^2]$, summed over all independent reflections.

[b] For the highest resolution shell in parenthesis.

[c] Represents approximately 5% of the data.

[d] Based on R_{free}.

Parameter	W434F			
	G2SG-OMe	4NP-G3SG	$(G4SG4O)_3$	G6SG-OMe
X-Ray source				
Wavelength	0.9537	0.9537	0.9537	0.9537
Space group	$P4_32_12$	$P4_32_12$	$P4_32_12$	$P4_32_12$
Unit cell dimensions	a=b=100.72; c=180.83; α=β=γ=90º	a=b=100.77; c=180.17; α=β=γ=90º	a=b=100.61; c=181.39; α=β=γ=90º	a=b=100.61; c=180.38; α=β=γ=90º
Resolution range	87.99-1.90	87.95-1.76	87.98-1.66	87.87-1.89
Highest shell (Å)	1.95-1.90	1.80-1.76	1.70-1.66	1.94-1.89
Reflections	69,207	87,843	104,312	70,770
Redundancy	10	13.7	22	24.2
R_{merge} [a] (%)	18.4 (88.9) [b]	11.9 (84.7) [b]	8.2 (82.4) [b]	12.0 (84.5) [b]
$\langle I/\sigma(I)\rangle$	4.5 (3.3)	36.5 (3.1)	31.5(3.7)	36.8 (2.1)
Completeness (%)	98.6 (79.6) [b]	99.9 (98.3) [b]	99.7 (99.6) [b]	99.7 (96.5) [b]
$CC_{1/2}$	0.835	0.997	0.996	0.997
Refinement statistics				
R_{work} (%)	16.0 (32.5) [b]	14.4 (17.7) [b]	14.8 (30.5) [b]	13.3 (18.1) [b]
R_{free} [c] (%)	20.7 (34.8) [b]	17.7 (21.7) [b]	18.1 (31.9) [b]	16.5 (22.2) [b]
No. of atoms (protein)	4,615	4,600	4,645	4,596
(sugar ligands)	25	23	34	25
(waters)	504	506	670	602
Mean B-value (Å)	32.44	21.79	25.75	25.19
Rms bonds (Å), angles (º)	0.020; 2.173	0.028; 2.658	0.030; 2.668	0.026; 2.308
Overall coordinate error [d] (Å)	0.112	0.078	0.071	0.087
Ramachandran plot statistics				
Residues	606	591	606	595
Most favoured region	88.8	90.2	89.4	90.7
Allowed regions	10.8	9.3	10.2	9.1
Disallowed region	0.4	0.4	0.4	0.2

[a] $R_{merge} = 100\ [\sum(I_i-\langle I\rangle)^2/\sum I_i^2]$, summed over all independent reflections.

[b] For the highest resolution shell in parenthesis.

[c] Represents approximately 5% of the data.

[d] Based on R_{free}.

Parameter	W434H			
	G2SG-OMe	4NP-G3SG	G4SG-OMe	G6SG-OMe
X-Ray source				
Wavelength	0.9537	0.9537	0.9537	0.9537
Space group	$P4_32_12$	$P4_32_12$	$P4_32_12$	$P4_32_12$
Unit cell dimensions	a=b=100.21; c=182.12; α=β=γ=90°	a=b=100.39; c=180.04; α=β=γ=90°	a=b=100.27; c=181.79; α=β=γ=90°	a=b=100.13; c=181.20; α=β=γ=90°
Resolution range	87.8-1.92	87.71-1.98	87.80-1.95	87.64-1.99
Highest shell (Å)	1.97-1.92	2.03-1.98	2.00-1.95	2.04-1.99
Reflections	67,572	61,724	64,595	60,320
Redundancy	29	29	29	28
R_{merge} [a] (%)	10.9 (88.1) [b]	13.2 (80.0) [b]	12.0 (84.7) [b]	14.5 (85.6) [b]
$\langle I/\sigma(I)\rangle$	36.9 (6.5) [b]	31.6 (5.9) [b]	32.0 (6.0) [b]	26.8 (5.5) [b]
Completeness (%)	99.9 (98.5) [b]	99.8 (97.8) [b]	99.9 (98.0) [b]	99.7 (95.8) [b]
$CC_{1/2}$	0.997	0.996	0.996	0.994
Refinement statistics				
R_{work} (%)	14.6 (17.4) [b]	14.4 (16.0) [b]	14.4 (17.2) [b]	15.7 (18.8) [b]
R_{free} [c] (%)	18.2 (22.5) [b]	17.7 (18.5) [b]	17.8 (23.7) [b]	19.4 (23.1) [b]
No. of atoms (protein)	4,607	4,640	4,631	4,598
(sugar ligands)	24	23	24	25
(waters)	681	483	621	491
Mean B-value (Å)	23.05	23.32	26.06	26.60
Rms bonds (Å), angles (°)	0.023; 2.232	0.026; 2.478	0.025; 2.315	0.023; 2.184
Overall coordinate error [d] (Å)	0.101	0.105	0.102	0.115
Ramachandran plot statistics				
Residues	604	591	606	606
Most favoured region	89.2	90.2	90.2	87.8
Allowed regions	10.7	9.4	9.4	11.8
Disallowed region	0.2	0.4	0.4	0.4

[a] $R_{merge} = 100\ [\sum(I_i - \langle I\rangle)^2/\sum I_i^2]$, summed over all independent reflections.

[b] For the highest resolution shell in parenthesis.

[c] Represents approximately 5% of the data.

[d] Based on R_{free}.

Parameter	W434Y			
	G2SG-OMe	4NP-G3SG	G4SG-OMe	G6SG-OMe
X-Ray source				
Wavelength	0.9537	0.9537	0.9537	0.9537
Space group	$P4_32_12$	$P4_32_12$	$P4_32_12$	$P4_32_12$
Unit cell dimensions	a=b=100.46; c=182.89; α=β=γ=90º	a=b=100.47; c=179.81; α=β=γ=90º	a=b=100.06; c=181.73; α=β=γ=90º	a=b=100.71; c=181.01; α=β=γ=90º
Resolution range	87.94-2.01	87.71-2.25	87.65-2.03	88.01-1.82
Highest shell (Å)	2.06-2.01	2.30-2.25	2.08-2.03	1.87-1.82
Reflections	59,659	42,365	56,607	79,565
Redundancy	29	29	28	29
R_{merge} [a] (%)	12.2 (85.2) [b]	16.0 (87.5) [b]	12.7 (86.0) [b]	9.3 (84.7) [b]
$<I/\sigma(I)>$	33.4 (6.0) [b]	26.4 (5.3) [b]	29.5 (5.8) [b]	38.2 (6.4) [b]
Completeness (%)	99.73 (97.5) [b]	99.8 (96.9) [b]	98.6 (80.1) [b]	99.9 (98.9) [b]
$CC_{1/2}$	0.996	0.994	0.995	0.997
Refinement statistics				
R_{work} (%)	15.8 (18.7) [b]	16.1 (19.6) [b]	15.1 (19.3) [b]	13.9 (16.7) [b]
R_{free} [c] (%)	20.2 (24.6) [b]	21.4 (30.0) [b]	19.4 (27.0) [b]	16.8 (21.3) [b]
No. of atoms (protein)	4,623	4,592	4,611	4,636
(sugar ligands)	24	23	23	24
(waters)	528	330	477	569
Mean B-value (Å)	27.53	27.51	27.46	22.74
Rms bonds (Å), angles (º)	0.021; 2.134	0.018; 1.994	0.022; 2.242	0.029; 2.606
Overall coordinate error [d] (Å)	0.123	0.169	0.122	0.080
Ramachandran plot statistics				
Residues	606	593	606	598
Most favoured region	90.8	88.0	89.2	91.0
Allowed regions	8.8	11.4	10.4	8.6
Disallowed region	0.4	0.6	0.4	0.4

[a] $R_{merge} = 100 [\sum(I_i-<I>)^2/\sum I_i^2]$, summed over all independent reflections.

[b] For the highest resolution shell in parenthesis.

[c] Represents approximately 5% of the data.

[d] Based on R_{free}.

Supplementary Table 7 The most populated conformational states of the β-D-glucopyranose rings of S-linked β-D-glucosides in the -1 and +1 subsites in crystal structures of WT and mutant HvExoI, and S-linked and O-linked β-D-glucosides after cMD simulations (median).

	β-D-Glucosides			
	2-linked [a]	3-linked [a]	4-linked [a]	6-linked [a]
S-Linked β-D-glucosides in crystal structures (-1 subsite)	$^{4}C_{1}$	$^{4}H_{3}$	$^{4}E/^{4}H_{3}$	$^{4}C_{1}$
S-Linked β-D-glucosides in crystal structures (+1 subsite)	$^{4}C_{1}$	$B^{1,4}$	$^{4}C_{1}$	$^{4}C_{1}$
S-Linked β-D-glucosides in crystal structures (-1 subsite) after cMD	92% ($^{4}C_{1}$); 7% ($^{4}H_{3}/^{4}E/^{4}H_{5}$)	95% ($^{4}C_{1}$); 3% ($^{4}H_{3}/^{4}E$); 1% ($^{1}S_{3}$)	43% ($^{4}C_{1}$); 35% ($^{4}H_{3}/^{4}E/^{4}H_{5}$); 17% ($^{1}S_{3}$)	80% ($^{4}C_{1}$); 11% ($^{4}H_{3}/^{4}E/^{4}H_{5}$); 7% ($^{1}S_{3}/^{1,4}B$)
S-Linked β-D-glucosides in crystal structures (+1 subsite) after cMD	$^{4}C_{1}$	53% ($^{4}C_{1}$); 39% ($^{2}S_{O}$)	>90% $^{4}C_{1}$	$^{4}C_{1}$
O-Linked β-D-glucosides after cMD (-1 subsite) [b]	93% ($^{4}C_{1}$); 5% ($^{4}H_{3}/^{4}E/^{4}H_{5}$)	63% ($^{4}C_{1}$); 32%($^{4}H_{5}/^{4}E$)	67% ($^{4}C_{1}$); 27% ($^{4}H_{3}/^{4}E/^{4}H_{5}$)	88% ($^{4}C_{1}$); 10% ($^{4}H_{3}/^{4}E/^{4}H_{5}$)
O-Linked β-D-glucosides after cMD (+1 subsite) [b]	>90% $^{4}C_{1}$	66% ($^{4}C_{1}$); 28% ($^{2}S_{O}$)	>90% $^{4}C_{1}$	>90% $^{4}C_{1}$

[a] Indicates % of a simulation time.

[b] O-Linked β-D-glycoside complexes were reconstructed from thio-analogue complexes.

Supplementary Table 8 List of primers to construct HvExoI mutants for heterologous expression in *P. pastoris*.

Mutant [a]	Number of mutations	Sequences (5' to 3') and notes [b]
W286A	Single	F:GGGTTTTGTTATTTCTGAT*GCT*GAAGGTATTGATAGAATTACTAC<br>R:GTAGTAATTCTATCAATACCTTC*AGC*ATCAGAAATAACAAAACCC
W286H	Single	F:GGGTTTTGTTATTTCTGAT*CAC*GAAGGTATTGATAGAATTACTAC<br>R:GTAGTAATTCTATCAATACCTTC*GTG*ATCAGAAATAACAAAACCC
W286F	Single	F:GGGTTTTGTTATTTCTGATT*TT*GAAGGTATTGATAGAATTACTAC<br>R:GTAGTAATTCTATCAATACCTTC*AA*AATCAGAAATAACAAAACCC
W286Y	Single	F:GGGTTTTGTTATTTCTGATT*AC*GAAGGTATTGATAGAATTACTAC<br>R:GTAGTAATTCTATCAATACCTTC*GT*AATCAGAAATAACAAAACCC
W434A	Single	F:GGTGGTTGGACTATTGAA*GCT*CAAGGAGATACTGGTAG<br>R:CTACCAGTATCTCCTTG*AGC*TTCAATAGTCCAACCACC
W434H	Single	F:GTGGTGGTTGGACTATTGAA*CAC*CAAGGAGATACTGGTAGAACTAC<br>R:GTAGTTCTACCAGTATCTCCTTG*GTG*TTCAATAGTCCAACCACCAC
W434F	Single	F:GTGGTGGTTGGACTATTGAAT*TT*CAAGGAGATACTGGTAGAAC<br>R:GTTCTACCAGTATCTCCTTG*AA*ATTCAATAGTCCAACCACCAC
W434Y	Single	F:GTGGTGGTTGGACTATTGAAT*AC*CAAGGAGATACTGGTAGAAC<br>R:GTTCTACCAGTATCTCCTTG*GT*ATTCAATAGTCCAACCACCAC
W286F/W434F	Double	Generated from W286F using W434F primers.
W286F/W434A	Double	Generated from W286F using W434A primers.

[a] All DNA fusions were sequenced in both directions and were found to be correct.

[b] F and R indicate forward and reverse primers, respectively. Mutations are highlighted in red italics.

Supplementary References

1. Streltsov, V. A., Luang, S., Peisley, A., Varghese, J. N., Ketudat Cairns, J. R. et al. Discovery of processive catalysis by an exo-hydrolase with a pocket-shaped active site. *Nat. Commun.* **10**, 2222 (2019).
2. Hrmova, M., DeGori, R., Smith, B. J., Fairweather, J. K., Driguez, H. et al. Structural basis for a broad specificity in higher plant β-D-glucan glucohydrolases. *Plant Cell* **14**, 1–22 (2002).
3. Hrmova, M., Varghese, J. N., DeGori, R., Smith, B. J., Driguez, H. et al. Catalytic mechanisms and reaction intermediates along the hydrolytic pathway of plant β-D-glucan glucohydrolase. *Structure* **9**, 1005–1016 (2001).
4. Lombard, V., Golaconda Ramulu, H., Drula, E., Coutinho, P. M. & Henrissat, B. The carbohydrate-active enzymes database (CAZy) in 2013. *Nucleic Acids Res.* **42D**, 490–495 (2014).
5. Edgar, R. C. MUSCLE: multiple sequence alignment with high accuracy and high throughput, *Nucleic Acids Res.* **32** 1792–1797 (2004).
6. Tamura, K., Stecher, G. & Kumar, S. MEGA 11: Molecular Evolutionary Genetics Analysis Version 11. *Mol. Biol. Evol.* **38**, 3022–3027 (2021).
7. Saitou, N. & Nei, M. The neighbor-joining method: A new method for reconstructing phylogenetic trees. *Mol. Biol. Evol.* **4**, 406-425 (1987).
8. Nei, M. & Kumar, S. Molecular Evolution and Phylogenetics. Oxford University Press, New York. (2000).
9. Contour-Galcera, M. O., Guillot, J. -M., Ortiz-Mellet, C., Pflieger-Carrara, F., Defaye, J. et al. Synthesis of sulfur-linked analogues of nigerose, laminarabiose, laminaratriose, gentiobiose, gentiotriose, and laminaran trisaccharide Y. *Carbohydr. Res*. **281**, 99–118 (1996).
10. Stick, R. V. & Stubbs, K. A. From glycoside hydrolases to thioglycoligases: The synthesis of thioglycosides. *Tetrahedron Asymmetry* **16**, 321–335 (2005).
11. Moreau, V., Norrild, J. C. & Driguez, H. Synthesis of methyl 4-thio-β-cellobioside. A reinvestigation. *Carbohydr. Res.* **300**, 271–277 (1997).
12. Hrmova, M. & Fincher, G. B. Barley β-D-glucan exohydrolases. Substrate specificity and kinetic properties. *Carbohydr. Res*. **305**, 209–221 (1998).
13. Hrmova, M. & Fincher, G. B. Purification and properties of three (1,3)-β-D-glucanase isoenzymes from young leaves of barley (*Hordeum vulgare*). *Biochem. J.* **289**, 453–461 (1993).
14. Fersht, A. Structure and Mechanism in Protein Science, pp 1–631, W. H. Freeman and Co., New York.Fersht, (1999).
