# Supplementary material for: The evolutionary advantage of an aromatic clamp in plant family 3 glycoside exo-hydrolases
Source: Nat Commun. 2022 Sep 23;13:5577. doi: 10.1038/s41467-022-33180-5 (PMC9508125; doi:10.1038/s41467-022-33180-5)
Supplement: Supplementary file 3 — Description of Additional Supplementary Files [file 41467_2022_33180_MOESM3_ESM.pdf]

Description of Additional Supplementary Files

File name: Supplementary Movie 1

Description: Molecular animation of sequences of events in WT HvExoI after the laminarihexaose substrate is hydrolysed into Glc and laminaripentaose (L5). Here, the Glc product adjusts its binding patterns and traverses from the -1 subsite through rotations of Arg158, Tyr253, Asp285, Glu491 sidechains, and associated backbone atoms into the autonomous and transient lateral cavity, from where it advances through the *ad-hoc* formed aperture into bulk solvent. These are key events of path 1 of substrate-product assisted processive catalysis. The video was prepared in Chimera (Pettersen, E. F. et al. *J. Comput. Chem.* 25, 1605–1612; 2004) using the HD Movie Maker tool.

File name: Supplementary Movie 2

Description: Molecular animation of sequences of events in the W434H mutant of HvExoI after the laminarihexaose substrate is hydrolysed into Glc and laminaripentaose (L5). Here, the Glc product adjusts its binding patterns and traverses from the -1 subsite through rotations of Arg158, Tyr253, Asp285, Glu491 sidechains, and associated backbone atoms into the autonomous and transient lateral cavity, from where it advances through the *ad-hoc* formed aperture into bulk solvent. These are key events of path 1 of substrate-product assisted processive catalysis. The video was prepared as described in the legend for Supplementary Movie 1.

File name: Supplementary Movie 3

Description: Molecular animation of sequences of events in the W434A mutant of HvExoI after the laminarihexaose substrate is hydrolysed into Glc and laminaripentaose (L5). Here, the Glc product diffuses through the preformed opening resulting from the W434A mutation. In this Glc egress path, the toll-like Arg158-Asp285-Glu491 barrier, Tyr253, and the Arg291-Glu220 salt bridge are not involved. These are key events of path 2, where substrate-product assisted processive catalysis does not take place. The video was prepared as described in the legend for Supplementary Movie 1.
