# Supplementary material for: The evolutionary advantage of an aromatic clamp in plant family 3 glycoside exo-hydrolases
Source: Nat Commun. 2022 Sep 23;13:5577. doi: 10.1038/s41467-022-33180-5 (PMC9508125; doi:10.1038/s41467-022-33180-5)
Supplement: Supplementary file 7 — Reporting Summary [file 41467_2022_33180_MOESM7_ESM.pdf]

nature portfolio

Corresponding author(s): Maria Hrmova

Last updated by author(s): 20 July 2022

Reporting Summary

Nature Portfolio wishes to improve the reproducibility of the work that we publish. This form provides structure for consistency and transparency in reporting. For further information on Nature Portfolio policies, see our Editorial Policies and the Editorial Policy Checklist.

Statistics

For all statistical analyses, confirm that the following items are present in the figure legend, table legend, main text, or Methods section.

n/a	Confirmed	
☐	☒	The exact sample size (*n*) for each experimental group/condition, given as a discrete number and unit of measurement
☒	☐	A statement on whether measurements were taken from distinct samples or whether the same sample was measured repeatedly
☒	☐	The statistical test(s) used AND whether they are one- or two-sided<br>*Only common tests should be described solely by name; describe more complex techniques in the Methods section.*
☒	☐	A description of all covariates tested
☒	☐	A description of any assumptions or corrections, such as tests of normality and adjustment for multiple comparisons
☐	☒	A full description of the statistical parameters including central tendency (e.g. means) or other basic estimates (e.g. regression coefficient) AND variation (e.g. standard deviation) or associated estimates of uncertainty (e.g. confidence intervals)
☒	☐	For null hypothesis testing, the test statistic (e.g. *F*, *t*, *r*) with confidence intervals, effect sizes, degrees of freedom and *P* value noted<br>*Give P values as exact values whenever suitable.*
☒	☐	For Bayesian analysis, information on the choice of priors and Markov chain Monte Carlo settings
☒	☐	For hierarchical and complex designs, identification of the appropriate level for tests and full reporting of outcomes
☒	☐	Estimates of effect sizes (e.g. Cohen's *d*, Pearson's *r*), indicating how they were calculated

Our web collection on statistics for biologists contains articles on many of the points above.

Software and code

Policy information about availability of computer code

Data collection	All software applications used in data collection at the Australian Synchrotron or on an in-house Rigaku MicroMax-007HF with a second-generation true microfocus rotating anode generator, refinement (REFMAC, MIFIT and Coot) and validation (PROCHECK) are described and relevant references are cited.
Data analysis	Software applications for crystal structure determination described in Methods with associated references were used without code modifications. For analyses of classical molecular dynamics and Gaussian accelerated MD simulations, and GPathFinder calculations, we used in-house scripts, which are available on GitHub at https://github.com/insilichem/utils/ and https://github.com/roviralab/utils.

For manuscripts utilizing custom algorithms or software that are central to the research but not yet described in published literature, software must be made available to editors and reviewers. We strongly encourage code deposition in a community repository (e.g. GitHub). See the Nature Portfolio guidelines for submitting code & software for further information.

Data

Policy information about availability of data

All manuscripts must include a data availability statement. This statement should provide the following information, where applicable:

- Accession codes, unique identifiers, or web links for publicly available datasets
- A description of any restrictions on data availability
- For clinical datasets or third party data, please ensure that the statement adheres to our policy

The atomic coordinates and structure factors were deposited in the Protein Data Bank (www.pdb.org) with the following PDB accessions: WT HvExoI, and in complex with 4NP-G3SG, and (G4SG4O)3 are 6JG2, and 6JG1, respectively. The PDB accession of W286A in complex with G6SG-OMe is 6JG6, and W286F in complex with G2OG-OMe, 4NP-G3SG, and G6SG-OMe are 6JG7, 6JGA, and 6JGB. The PDB accession of W286Y in complex with Glc, and G6SG-OMe are 6JGC and 6JGD. The PDB accession of W434A in complex with Glc, G2SG-OMe, 4NP-G3SG3OG, (G4SG4O)3, and G6SG-OMe are 6KUF, 6JGE, 6LIJ, 6LBB, and 6K6V. The PDB accession of W434F in complex with G2SG-OMe, 4NP-G3SG, (G4SG4O)3, and G6SG-OMe are 6JGG, 6LC5, 6JGK, and 6LBV. The PDB accession of W434H in complex with G2SG-OMe, 4NP-G3SG, G4SG-OMe, and G6SG-OMe are 6JGL, 6JGN, 6JGO, and 6JGP. The PDB accession of W434Y in complex with G2sG-OMe, 4NP-G3SG, G4SG-OMe, and G6SG-OMe are 6JGQ, 6JGR, 6JGS, and 6JGT. All crystal structures were released and are available in the Protein Data Bank.

Other structural files from PDB include native WT, recombinant WT, the recombinant form in complex with G2SG-OMe and G6SG-OMe with respective PDB accessions of 3WLH, 3WLO, 6MD6 and 3WLP.

Source data are provided with this paper.

Human research participants

Policy information about studies involving human research participants and Sex and Gender in Research.

Reporting on sex and gender	Not applicable.
Population characteristics	Not applicable.
Recruitment	Not applicable.
Ethics oversight	Not applicable.

Note that full information on the approval of the study protocol must also be provided in the manuscript.

Field-specific reporting

Please select the one below that is the best fit for your research. If you are not sure, read the appropriate sections before making your selection.

☒ Life sciences ☐ Behavioural & social sciences ☐ Ecological, evolutionary & environmental sciences

For a reference copy of the document with all sections, see nature.com/documents/nr-reporting-summary-flat.pdf

Life sciences study design

All studies must disclose on these points even when the disclosure is negative.

Sample size	For enzyme activity measurements, sample sizes were determined empirically using our extended experience using similar assays, as described in several studie.<br><br>The relevant References are:<br>(i) Streltsov, V. A., Luang, S., Peisley, A., Varghese, J. N., Ketudat Cairns, J. R. et al. Discovery of processive catalysis by an exo-hydrolase with a pocket-shaped active site. Nat. Commun. 10, 2222 (2019).<br><br>(ii) Luang, S., Hrmova, M. & Ketudat Cairns, J. R. High-level expression of barley β-D-glucan exohydrolase HvExoI from a codon-optimized cDNA in Pichia pastoris. Prot. Exp. Purif. 73, 90–98 (2010).<br><br>(iii) 60. Luang, S., Ketudat Cairns, J. R., Streltsov, V. A. & Hrmova, M. Crystallisation of wild-type and variant forms of a recombinant β-D-glucan glucohydrolase from barley (Hordeum vulgare L.) by macroseeding with wild-type native microcrystals and preliminary X-ray analysis. Int. J. Mol. Sci. 11, 2759–2769 (2010).
Data exclusions	No data were excluded from experiments.
Replication	Apparent first-order rate kinetic parameters (KM and kcat) were determined with (1,3;1,6)-β-D-glucan (laminarin), barley (1,4;1,3)-β-D-glucan, laminaribiose, cellobiose, and 4NP-Glc with substrate concentrations ranging between 0.3-3 times KM values in duplicate. Apparent inhibition

	constants of enzyme:inhibitor complexes (Ki) using the 4NP-Glc substrate were determined at six concentrations with G2SG-OMe, 4NP-G3SG, G4SG-OMe, G4SG4OG4SG4OG4SG, and G6SG-OMe at 0.4-3 times the Ki values in duplicate.
Randomization	Covariates were not used in this study.
Blinding	Investigators were not blinded.

Reporting for specific materials, systems and methods

We require information from authors about some types of materials, experimental systems and methods used in many studies. Here, indicate whether each material, system or method listed is relevant to your study. If you are not sure if a list item applies to your research, read the appropriate section before selecting a response.

Materials & experimental systems

n/a	Involved in the study
☒	☐ Antibodies
☒	☐ Eukaryotic cell lines
☒	☐ Palaeontology and archaeology
☒	☐ Animals and other organisms
☒	☐ Clinical data
☒	☐ Dual use research of concern

Methods

n/a	Involved in the study
☒	☐ ChIP-seq
☒	☐ Flow cytometry
☒	☐ MRI-based neuroimaging

March 2021
